# Systematic analysis of the expression and prognostic value of ITPR1 and correlation with tumor infiltrating immune cells in breast cancer

**DOI:** 10.1186/s12885-022-09410-w

**Published:** 2022-03-21

**Authors:** Bing Han, Fang Zhen, Xiu-Shuang Zheng, Jing Hu, Xue-Song Chen

**Affiliations:** 1 Department of Breast Medical Oncology, Harbin Medical University Cancer Hospital, 150 Haping Road, Harbin, 150040 China; 2 Department of Reproductive Medicine, The First Affiliated Hospital of Harbin Medical University, 23 Youzheng Street, Harbin, 150001 China

**Keywords:** ITPR1, Breast cancer, Immune infiltration, Prognostic biomarker, Prognosis

## Abstract

**Background:**

ITPR1 is a key gene for autophagy, but its biological function is still unclear, and there are few studies on the correlation between ITPR1 gene expression and the occurrence and development of breast cancer.

**Methods:**

Analyze the expression of ITPR1 through online databases such as Oncomine and TIMER. Kaplan–Meier plotter and other databases were used to evaluate the impact of ITPR1 on clinical prognosis. The expression of ITPR1 in analysis of 145 cases of breast cancer and 30 cases of adjacent normal tissue was detected by Immunohistochemistry. Statistical analysis was used to evaluate the clinical relevance and prognostic significance of abnormally expressed proteins. And the Western Blot was used to detect the expression of ITPR1 between breast cancer tissues and cells. The TIMER database studied the relationship between ITPR1 and cancer immune infiltration. And used the ROC plotter database to predict the response of ITPR1 to chemotherapy, endocrine therapy and anti-HER2 therapy in patients with breast cancer.

**Results:**

Compared with normal breast samples, ITPR1 was significantly lower in patients with breast cancer. And the increased expression of ITPR1 mRNA was closely related to longer overall survival (OS), distant metastasis free survival (DMFS), disease specific survival (DSS) and relapse free survival (RFS) in breast cancer. And the expression level of ITPR1 was higher in patients treated with chemotherapy than untreated patients. In addition, the expression of ITPR1 was positively correlated with related gene markers of immune cells in different types of breast cancer, especially with BRCA basal tissue breast cancer.

**Conclusion:**

ITPR1 was lower expressed in breast cancer. The higher expression of ITPR1 suggested favorable prognosis for patients. ITPR1 was related to the level of immune infiltration, especially in BRCA-Basal patients. All research results indicated that ITPR1 might affect breast cancer prognosis and participate in immune regulation. In short, ITPR1 might be a potential target for breast cancer therapy.

**Supplementary Information:**

The online version contains supplementary material available at 10.1186/s12885-022-09410-w.

## Introduction

Breast cancer is the number one killer of women's health in the world. In recent years, the increasing morbidity and mortality have become a major hidden danger to the world's health problems [1]. The latest data show that breast cancer has officially replaced lung cancer as the world's largest cancer [2]. At present, the treatment methods for breast cancer usually include surgery, chemotherapy, radiation therapy, targeted therapy and endocrine therapy. Although these treatments can improve the prognosis of breast cancer to a certain extent, the survival of some patients is still poor [3, 4]. Therefore, looking for new prognostic indicators and clarifying the pathogenesis of breast cancer are of great significance for providing new opportunities for early detection and early treatment and reducing the mortality and recurrence rate of breast cancer.

Inositol 1, 4, 5-trisphosphate receptor type 1 (ITPR1), located on chromosome 3, is a member of the IP3R family, involved three distinct IP3R type in mammals [5, 6]. ITPR1 is an intracellular Ca^2+^ release channel, and its opening requires the combination of two intracellular messengers IP3 and Ca^2+^. Many physiological processes are related to the increase of intracellular Ca^2+^ concentration, either through the absorption of Ca^2+^ in the extracellular environment or the release of calcium ions in the intracellular environment. The second messenger 1,4,5-triphosphate (IP3) is the product of phosphatidylinositol 4,5-bisphosphate hydrolyzed by G protein-coupled receptor/phospholipase C (PLC-β) or tyrosine kinase receptor/PLC-γ signaling pathway activates the Ca^2+^ release of the endoplasmic reticulum (ER) [7]. IP3 acts by binding to the membrane-associated IP3 receptor (IP3R) [8, 9]. The binding of IP3 to the receptor increases its sensitivity to Ca^2+^, and only after it binds with Ca^2+^ can enter the cytoplasm. It is worth noting that Ca^2+^ has a biphasic effect on IP3R, low Ca^2+^ concentration can stimulate IP3R, and high Ca^2+^ concentration can inhibit IP3R [10, 11].

The most widely studied IP3R is type 1 (ITPR1), and high levels of ITPR1 are found in Purkinje cells of the cerebellum of the central nervous system [12, 13]. The defect of ITPR1 is the cause of spinocerebellar ataxia type 15 (SCA15) [14]. The interacts with TMEM173 and ITPR1 could promote the release of endoplasmic reticulum calcium, leading to subsequent F3 release and coagulation activation in patients with sepsis [15]. In bladder cancer, the overexpression of ITPR1 in drug-resistant cells could induce cell apoptosis and increase sensitivity to cisplatin [16].

In recent years, studies have find that ITPR1 is a pivotal gene for autophagy [17]. Autophagy was a process of engulfing its own cytoplasmic proteins and turning them into autophagy lysosomes to degrade the contents it contains. Autophagy played a role in starvation response. ITPR1 induced the release of Ca^2+^ and promoted ATP synthesis in the non-starvation state, thereby inhibiting AMPK activity and inhibiting autophagy. In the starvation state, Ca^2+^ activity would activate the autophagy pathway and promote the formation of autophagy. The regulation of ITPR1 is closely related to Bcl2 and Beclin1, which are generally in a combined state. When starved, Bcl2 and Beclin1 became dissociated, and then Beclin1 formed a complex with ITPR1 activating and promoting the production of autophagy [18–20]. ITPR1 participated in autophagy induced by NK cells and reduced the killing effect of cytokines secreted by NK cells on kidney cancer [21]. ITPR1 was a new target of HIF-2α, which protected kidney cancer cells from NK-mediated lysis by inducing NK-mediated autophagy [22]. As an autophagy gene, ITPR1 was down-regulated in head and neck tumor and esophageal cancer [23, 24]. However, the systematic analysis of ITPR1 in breast cancer is still rare, and the relationship between the expression of ITPR1 and the survival of breast cancer patients is unclear.

This study comprehensively studied the expression of ITPR1 in patients with breast cancer. and its relationship with prognosis in online databases such as Oncomine, GEPIA, and Kaplan–Meier plotter. And confirmed by immunohistochemistry method. In addition, the TIMER database was used to analyze the correlation between ITPR1 and tumor infiltrating immune cells. The results of this study clarify the mechanism of ITPR1 gene and its prognostic significance in treatment, and provide the potential relationship and mechanism of ITPR1 and tumor immune interaction.

## Methods

### ONCOMINE database

Oncomine (https://www.oncomine.org) is currently the world's largest oncogene chip database and integrated data mining platform. It had the most complete cancer mutation profile, gene expression data and related clinical information, which could be used to discover new biomarkers or new therapeutic targets [25]. The mRNA expression level of ITPR1 gene in pan-carcinoma was analyzed by Oncomine, and the mRNA level of ITPR1 between normal and breast cancer tissues was compared (setting parameters were twofold change, *P* value ≤ 0.01 and top 10% gene rank).

### GEPIA

GEPIA (http://gepia.cancer-pku.cn/) was the dynamic analysis of gene expression profiling data, a public database for cancer and normal gene expression profiling, filling the gap in cancer genomics big data information. Including 9736 tumors from TCGA and GTEx projects and RNA sequencing expression data of 8587 normal samples [26]. GEPIA (Gene Expression Profiling Interactive Analysis) analyzed the expression level of ITPR1 in different tumor types, and compared the expression level of ITPR1 in normal and breast cancer tissues(setting parameters were |Log2FC|= 1, *P* value ≤ 0.01).

### TNMplot database

TNMplot database (http://www.tnmplot.com) used gene arrays from the National Center for Biotechnology Information (NCBI-GEO) Gene Expression Comprehensive Database or RNA-seq from the Cancer Genome Atlas (TCGA) to generate effective therapeutic application research Data generated from the treatment (target) and genotype tissue expression (GTEx) repository [27]. We used TNMplot database to verify the expression of ITPR1 in various cancers, and explored the expression of ITPR1 in normal breast, breast cancer and metastatic tissues.

### Breast cancer Gene-Expression Miner v4.5 (Bc-GenExMiner v4.5)

Bc-GenExMiner v4.5 (http://bcgenex.centregauducheau.fr/BC-GEM/GEM-Accueil.php?js=1) was a data mining tool that contains 36 published annotated genome data [28]. We used the expression module of Bc-GenExMiner v4.5 to analyze the expression level of ITPR1 in normal and breast cancer, and according to clinical standards (such as estrogen receptor (ER), progesterone receptor (PR), epidermal growth factor receptor 2 (HER2), nodular status, triple-negative status and basal-like status, lymph node status, Scarff-Bloom-Richardson classification (SBR), Nottingham prognostic index (NPI), etc.) to analyze the relationship between ITPR1 and breast cancer. In addition, we used correlated modules to analyze the relationship between ITPR1 and co-expressed genes.

### Human protein atlas

Human Protein Atlas (https://www.proteinatlas.org) was based on proteomics, transcriptomics and systems biology data, which could map tissues, cells, organs, etc. It not only includes tumor tissues, but also covers the protein expression of normal tissues [29]. The Human Protein Atlas database was used to analyze the expression of ITPR1 in breast cancer and normal tissues by immunohistochemistry.

### PrognoScan

PrognoScan (http://dna00.bio.kyutech.ac.jp/PrognoScan/index.html) integrated a large number of microarray data sets with prognostic information, including most tumor data, which could be used to analyze the relationship between gene expression and patient prognosis [30]. We used PrognoScan database to analyze the correlation between ITPR1 mRNA expression and survival of breast cancer patients (cox *P* value < 0.05).

### The Kaplan–Meier Plotter

Kaplan–Meier Plotter (http://kmplot.com/analysis/) was constructed based on gene chips and RNA-seq data from public databases such as GEO, EGA, and TCGA, and evaluated the impact of 54,675 genes on survival rates in 21 cancers. Meta-analysis and research, discovery and verification of survival-related molecular markers were carried out by integrating gene expression information and clinical prognostic value [31]. Kaplan–Meier Plotter database was used to analyze the survival correlation between ITPR1 mRNA expression and breast cancer patients and clinical molecular markers of breast cancer. (Set parameters as best cutoff, hazard ratio (HR) with 95% confidence intervals (CIs), log rank *P* value and JetSet best probe).

### ROC plotter

The ROC plotter (http://www.rocplot.org) was the first online transcriptome level verification tool for predicting biomarkers [32]. The ROC plotter is capable to link gene expression and response to therapy using transcriptome-level data of 3,104 breast cancer patients and 2,369 ovarian cancer patients. We used ROC plotter to predict the expression of breast cancer patients in response to chemotherapy, endocrine therapy, and anti-HER2 therapy. (Set the probe: ITPR1-203710_at, Response: Relapse-free survival at 5 years, Treatment: Endocrine therapy, Anti-HER2 therapy and Chemotherapy choose any, Settings: No outliers).

### Immunohistochemistry

Immunohistochemistry (IHC) was performed to detect the expression of ITPR1 in breast cancer. IHC method and criteria for judging results were referred to literature [33]. The antibodie was (Proteintech, 1:100) for ITPR1.

### Cell lines and cell culture

All breast cancer cell lines are from the Chinese Academy of Biochemistry and Cell Biology (Shanghai, China) and are regularly certified (Cellbio). The cells are preserved in Heilongjiang Cancer Institute (Harbin, China). These cells were cultured in DMEM, L15 or RPMI1640 medium in a humidified incubator with 10% fetal bovine serum and 1% penicillin–streptomycin, cultured at 37 °C with 5% CO2 or air.

### Western blot assay and antibodies

The lysed protein was electrophoresed on a 10% polyacrylamide gel (Sevenbio), and then transferred to the membrane at 300 mA for 5 h, blocked with skim milk for 1 h, and incubated overnight at 4 °C with the target antibody. On the second day, the secondary antibody corresponding to the target antibody was incubated for 1 h. The antibodie was (Proteintech, 1:100) for ITPR1.

### STRING

STRING (http://www.string-db.org) was a database for searching known and predicted protein interactions [34]. We used the STRING database to analyze the PPI network of ITPR1 and co-expressed genes (setting the parameters as Homo sapiens and combined score of > 0.4 was considered statistically significant). It also analyzed the functions of ITPR1, including Gene Ontology (GO) and Kyoto Encyclopedia of Genes and Genomes (KEGG). GO analysis focuses on the three areas of biological process (BP), cell composition (CC) and molecular function (MF). Only *P* Values < 0.05 were considered meaningful.

### TIMER database analysis

Tumor immune estimation resource (https://cistrome.shinyapps.io/timer/) used RNA-Seq expression profile data to detect the infiltration of immune cells in tumor tissues [35]. The database provided the infiltration status of 6 immune cells (B cells, CD4 + T cells, CD8 + T cells, Neutrphils, Macrophages and Dendritic cells) [36]. TIMER analyzed the expression level of ITPR1 in different tumor types, and analyzed the relationship between ITPR1 and 6 immune cells in breast cancer and its different types through gene modules. In addition, the relationship between ITPR1 and gene markers of tumor infiltrating immune cells in breast cancer and its different types was also explored.

### Statistical analysis

The analysis results were represented by HR and *P* or COX *P*-values of a log-rank test. The unpaired T-test was used to compare two means. The correlation of gene expression was evaluated by Spearman’s correlation and statistical significance. The absolute value of correlation is judged as follows: 0.30–0.40 “moderate,” 0.40–0.50 “strong”, and significance was defined as ***P < 0.001,**P < 0.01,*P < 0.05.

## Results

### Low expression of ITPR1 in patients with breast cancer

**Fig. 1:**
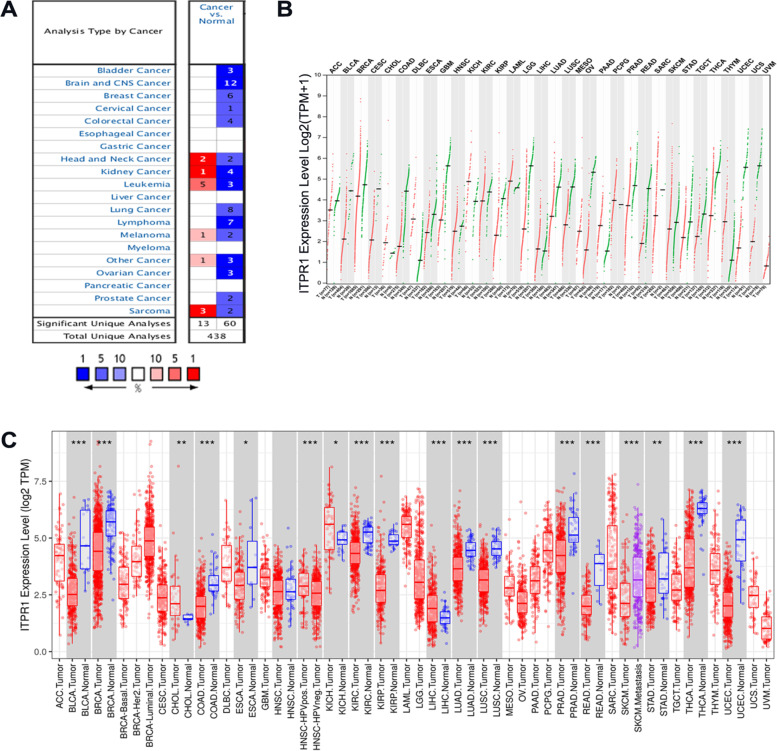
The expression of ITPR1 in distinct types of cancer diseases. **A** Expression of ITPR1 gene in 20 common tumors compared with paired normal tissues. Oncomine database was designed with fold change ≥ 2, *P* value ≤ 0.01 and gene rank ≥ top 10%. The graphic represents the numbers of datasets with statistically significant (*p* < 0.01) mRNA over-expression (red) or down-expression (blue) of ITPR11 (different types of cancer vs. corresponding normal tissue). **B** The Expression of ITPR1 in distinct types of cancer diseases (GEPIA). The differental methed choose Top 10 and use log2 (TPM + 1) for log-scale. **C** The Expression of ITPR1 in distinct types of cancer diseases (TIMER)

In order to explored the expression of ITPR1 and its unique prognosis, the Oncomine database was first used to detect the expression of ITPR1 in 20 kinds of common cancers. We found that the expression of ITPR1 gene is unstable in head and neck cancer, kidney cancer, leukemia, melanoma and sarcoma, but in bladder cancer, brain and central nervous system cancer, breast cancer, cervical cancer, colorectal cancer, lung cancer, lymphoma, Ovarian cancer and prostate cancer had lower expression (Fig. 1A). Next, we used the GEPIA dataset and the TIMER dataset to compare the expression of ITPR1 between tumor tissues and normal tissues (Fig. 1B, C). In addition, we used the TNMplot database to verify the expression of ITPR1 (Supplementary Fig. 1A). The results showed ITPR1 had lower expression in most tumor tissues than in normal tissues.

**Fig. 2:**
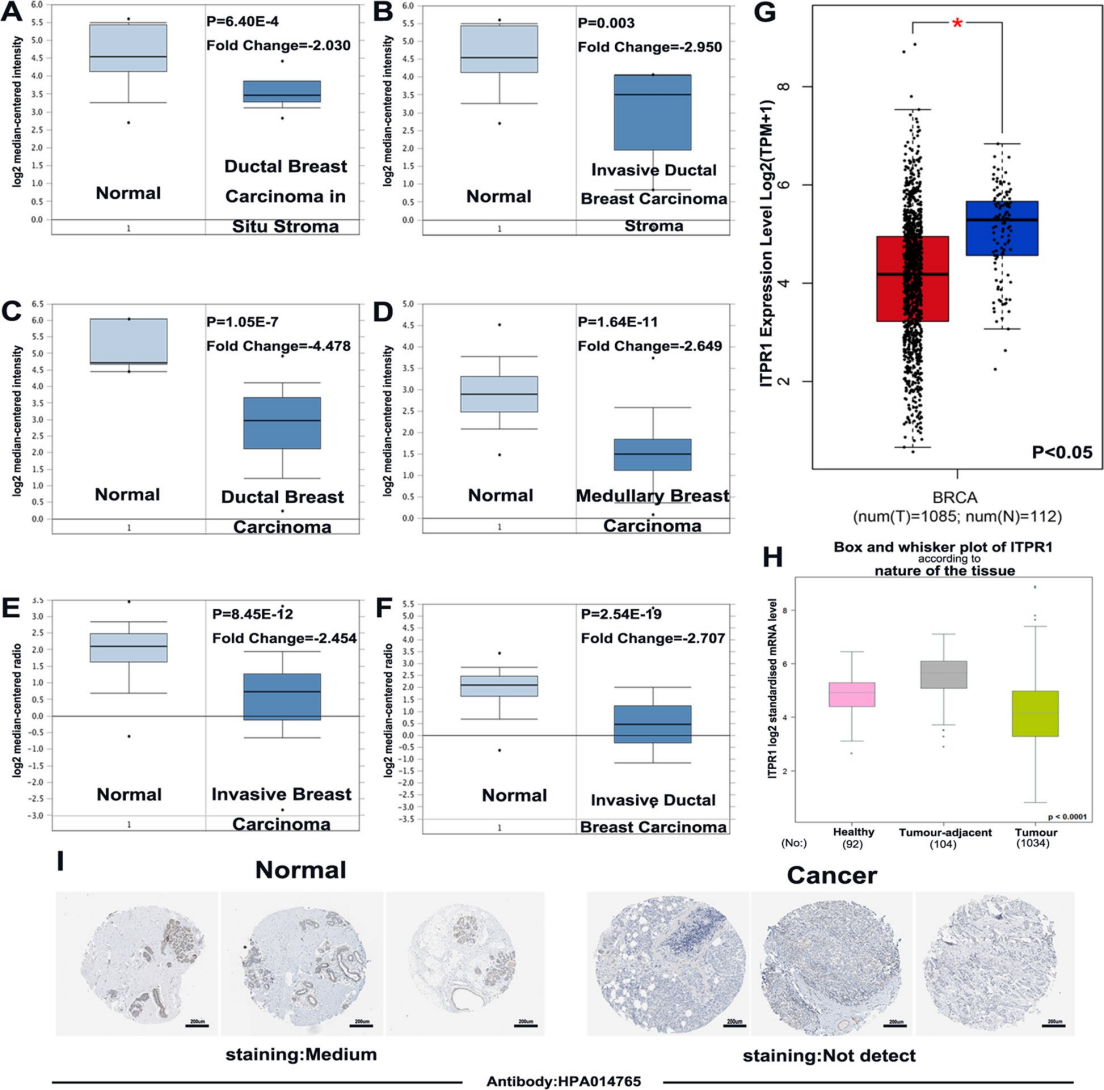
Box plots of normal and tumor differentially expression of ITPR1 gene in different subtypes of breast cancer. **A**-**F** Box plots of normal and tumor differentially expression of ITPR1 gene in different subtypes of breast cancer. **A** Ductal breast carcinoma in situ stroma (**B**) Invasive ductal breast carcinoma stroma (**C**) Ductal breast carcinoma (**D**) Medullary breast carcinoma (**E**) Invasive breast carcinoma (**F**) Invasive ductal breast carcinoma.(ONCOMINE). **G** Box plots of normal and tumor expression of ITPR1 gene in Breast Cancer. (GEPIA). **H** ITPR1 gene expression with box plots in patients with breast cancer. (Bc-GenExMiner v4.3) (**I**) Representative immunohistochemistry images of distinct ITPR1 in Breast Cancer tissues and normal tissues (Human Protein Atlas)

Compared with normal, the ITPR1 expression levels in ductal breast carcinoma in situ stroma, invasive ductal breast cancer stroma, ductal breast cancer, medullary breast cancer, invasive breast cancer and invasive ductal breast cancer were significantly reduced (Fig. 2A-F *P*= 6.40E-4, 0.003, 1.05E-7, 1.64E-11, 8.45E-12 and 2.54E-19, Supplementary Table 1). And in GEPIA and Bc-GenExMiner v4.3, the expression level of ITPR1 in breast cancer tissues is lower than normal tissues (Fig. 2G, H). In addition, the analysis of gene chip data and RNA-seq data through the TNMplot database showed that the expression level of ITPR1 in tumors and metastatic tissues was lower than normal tissues (Supplementary Fig. 1B, C).

**Fig. 3:**
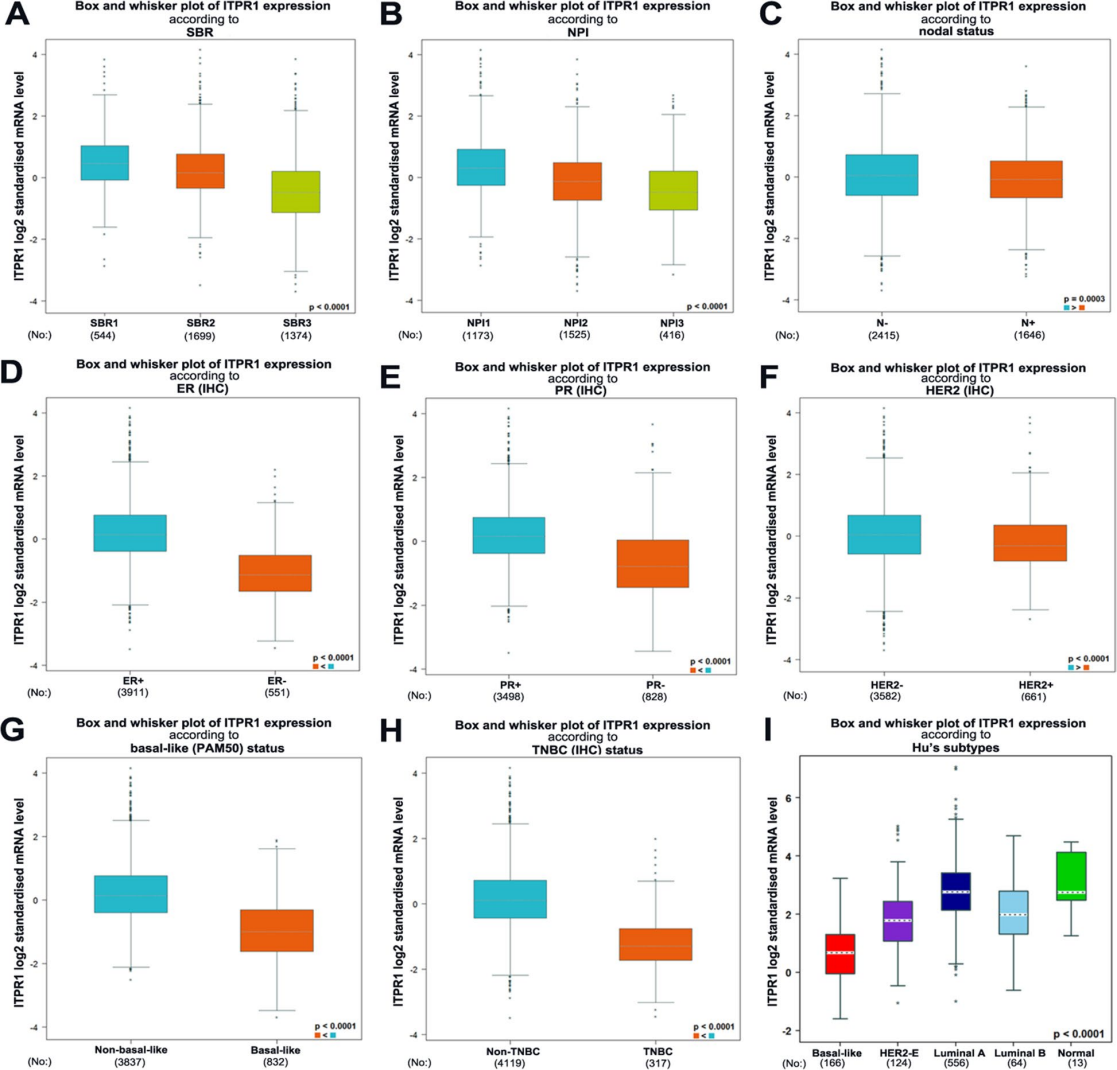
Bc-GenExMiner v4.3 to evaluate ITPR1 gene expression with box plots according to clinical parameters in patients with breast cancer. **A** SBR grade (**B**) NPI (**C**) nodal status (**D**) ER (**E**) PR (**F**) HER-2 (**G**) basal-like status (**H**) triple-negative status (**I**) basal-like and triple-negative status

After verifying the mRNA expression of ITPR1 in breast cancer, we continued to explore the protein expression of ITPRI in breast cancer through the Human Protein Atlas. As we shown, ITPR1 was beyond detection in breast cancer tissues, but was moderately expressed in normal tissues (Fig. 2I). Taken together, our results indicate that ITPR1 is under-expressed in patients with breast cancer.

## The expression of ITPR1 in the clinical and pathological characteristics of breast cancer patients

Bc-GenExMiner v4.3 software was used to evaluate the expression of ITPR1 in clinical and pathological features in patients with breast cancer (Supplementary Table 2). The results showed that the expression of ITPR1 decreased with the increase of SBR grade and NPI grade of breast cancer patients (Fig. 3A-B, *P* < 0.0001). Compared with negative lymph nodes, the expression of ITPR1 was reduced in patients with positive lymph nodes (Fig. 3C, *P* = 0.0003). ITPR1 was highly expressed in ER and PR-positive breast cancer patients (Fig. 3D-E, *P* < 0.0001). The expression was lower in patients with HER2-positive breast cancer (Fig. 3F, *P* < 0.0001). In addition, the ITPR1 of patients with triple-negative and basal breast cancer was significantly lower than that of patients with non-triple-negative and non-basal breast cancer (Fig. 3G, H, I, *P* < 0.0001).

## The influence of ITPR1 expression on the prognosis of breast cancer

**Fig. 4:**
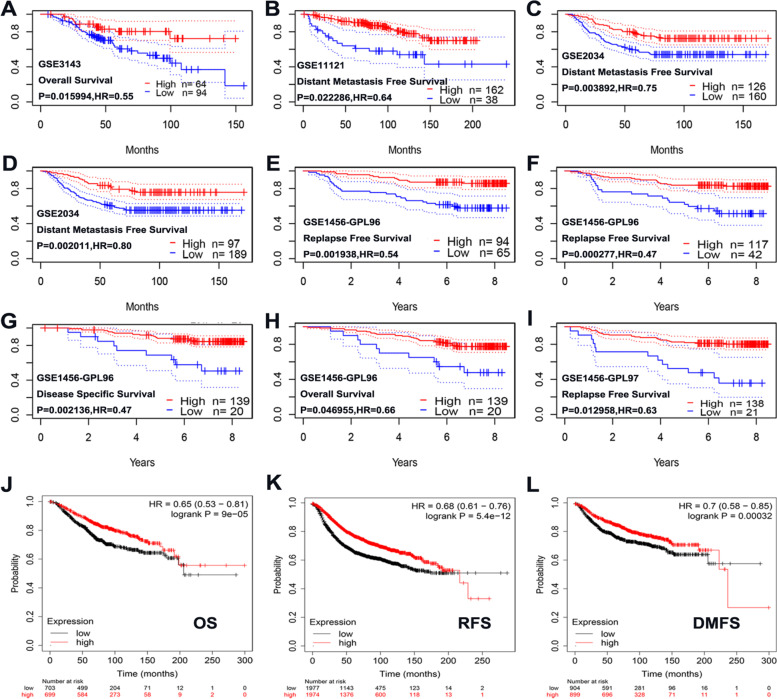
The prognostic value of mRNA level of ITPR1 in patients with breast cancer. **A**-**I** The survival curve of different datasets based on the expression of ITPR1 gene was used to analyze the prognostic value in breast cancer (PrognoScan). **A** Overall Survival (**B**) Distant Metastasis Free Survival (**C**) Distant Metastasis Free Survival (**D**) Distant Metastasis Free Survival (**E**) Relapse Free Survival (**F**) Relapse Free Survival (**G**) Disease Specific Survival (**H**) Overall Survival (**I**) Relapse Free Survival. **J**-**L** Prognostic value of mRNA expression of ITPR1 in patients with breast cancer (Kaplan–Meier Plotter). **J** Overall Survival (**K**) Relapse Free Survival (**L**) Distant Metastasis Free Survival

Using the survival meta-analysis software PrognoScan to draw survival curves with different survival information, breast cancer patients with ITPR1 (red) were positively correlated with overall survival, distant metastasis free survival, relapse free survival, disease-specific survival (Fig. 4A-I, Supplementary Table 3). In addition, we used the Kaplan–Meier plotter to verify the prognostic value of ITPR1 mRNA expression in patients with breast cancer. As shown in the figure, breast cancer patients with high levels of ITPR1 mRNA have high OS (HR = 0.65, 95% CI: 0.53–0.81, *P* = 9e-05), RFS (HR = 0.68, 95% CI: 0.61- 0.76, *P* = 5.4e-12) and DMFS (HR = 0.7, 95% CI: 0.58–0.85, *P* = 0.00032) (Fig. 4J-L, Supplementary Table 3). These results indicated that the expression of ITPR1 was significantly related to the prognosis of breast cancer patients, and may be used as a useful biomarker to predict the survival of breast cancer patients.

Besides, we further explored the mechanism by which ITPR1 expression affects the prognosis of breast cancer. First, the correlation between the expression of ITPR1 and clinical variables was analyzed by KM plotter (Table 1). Specifically, the expression level of ITPR1 was related to basal (RFS HR = 1.3, *P* = 0.027), luminal A (OS HR = 0.57, *P* = 0.00056, RFS HR = 0.64, *P* < 0.0001), luminal B (RFS HR = 0.8, *P* = 0.032), HER2 (OS HR = 0.48 *P* = 0.011) disease subtypes were correlated. Then, we continued to explore the predictive value of ITPR1 expression level for the clinical treatment of breast cancer. Through ROC plotter analysis, the expression of ITPR1 did not change much in response to endocrine therapy or anti-HER2 therapy (Supplementary Figs. 2A and C). However, the expression level of ITPR1 in responders chemotherapy patients was higher than that in nonresponders chemotherapeutic patients (Supplementary Fig. 2E). The expression level of ITPR1 could predict the effect of chemotherapy, the AUC value was 0.589, *P* < 0.05 (Supplementary Fig. 2B, D, F). The above results suggested that the high expression of ITPR1 may affect the prognosis of breast cancer.

**Table 1:** Correlation of ITPR1 mRNA expression and clinicopathological factors in Breast cancer by Kaplan–Meier plotter database

Variables of breast cancer	Overall survival (*n* = 1880)			Relapse free survival (*n* = 4929)		
	**N**	**Hazard ratio**	***P*-value**	**N**	**Hazard ratio**	***P*-value**
ER						
Positive	**754**	**0.69 (0.5–0.94)**	**0.017**	**2633**	**0.66 (0.55–0.8)**	** < 0.0001**
Negative	**520**	**0.63 (0.41–0.96)**	**0.031**	1190	1.21 (0.97–1.5)	0.087
PR						
Positive	156	2.37 (0.9–6.25)	0.071	**926**	**0.56 (0.39–0.8)**	**0.0011**
Negative	**291**	**0.53 (0.29–0.98)**	**0.039**	**925**	**0.76 (0.59–0.99)**	**0.04**
HER2						
Positive	**420**	**0.6 (0.41–0.88)**	**0.0086**	882	0.86 (0.68–1.08)	0.19
Negative	**1459**	**0.58 (0.46–0.72)**	** < 0.0001**	**4047**	**0.62 (0.56–0.7)**	** < 0.0001**
Intrinsic subtype						
basal	404	0.63 (0.39–1.02)	0.058	**846**	**1.3 (1.03–1.65)**	**0.027**
luminal A	**794**	**0.57 (0.41–0.79)**	**0.00056**	**2277**	**0.64 (0.53–0.76)**	** < 0.0001**
luminal B	515	0.76 (0.52–1.11)	0.16	**1491**	**0.8 (0.66–0.98)**	**0.032**
HER2 +	**166**	**0.48 (0.27–0.86)**	**0.011**	315	0.74 (0.5–1.08)	0.12
Lymph node status						
Positive	**452**	**0.69 (0.48–0.99)**	**0.046**	**1656**	**0.67 (0.55–0.81)**	** < 0.0001**
Negative	**722**	**0.53 (0.37–0.78)**	**0.00087**	**2368**	**0.63 (0.54–0.75)**	** < 0.0001**
Grade						
1	175	0.52 (0.17–1.56)	0.23	**397**	**0.56 (0.31–1)**	**0.046**
2	**443**	**0.6 (0.4–0.9)**	**0.012**	**1177**	**0.61 (0.49–076)**	** < 0.0001**
3	586	0.85 (0.63–1.16)	0.31	**1300**	**1.27 (1.01–1.59)**	**0.041**
TP53 mutation						
Positive	130	0.73 (0.38–1.42)	0.36	188	0.63 (0.38–1.05)	0.071
Negative	**197**	**0.44 (0.23–0.81)**	**0.0068**	**273**	**0.61 (0.4–0.93)**	**0.021**

### Compared with normal breast tissue, ITPR1 is lower in breast cancer and is associated with prognosis

**Fig. 5:**
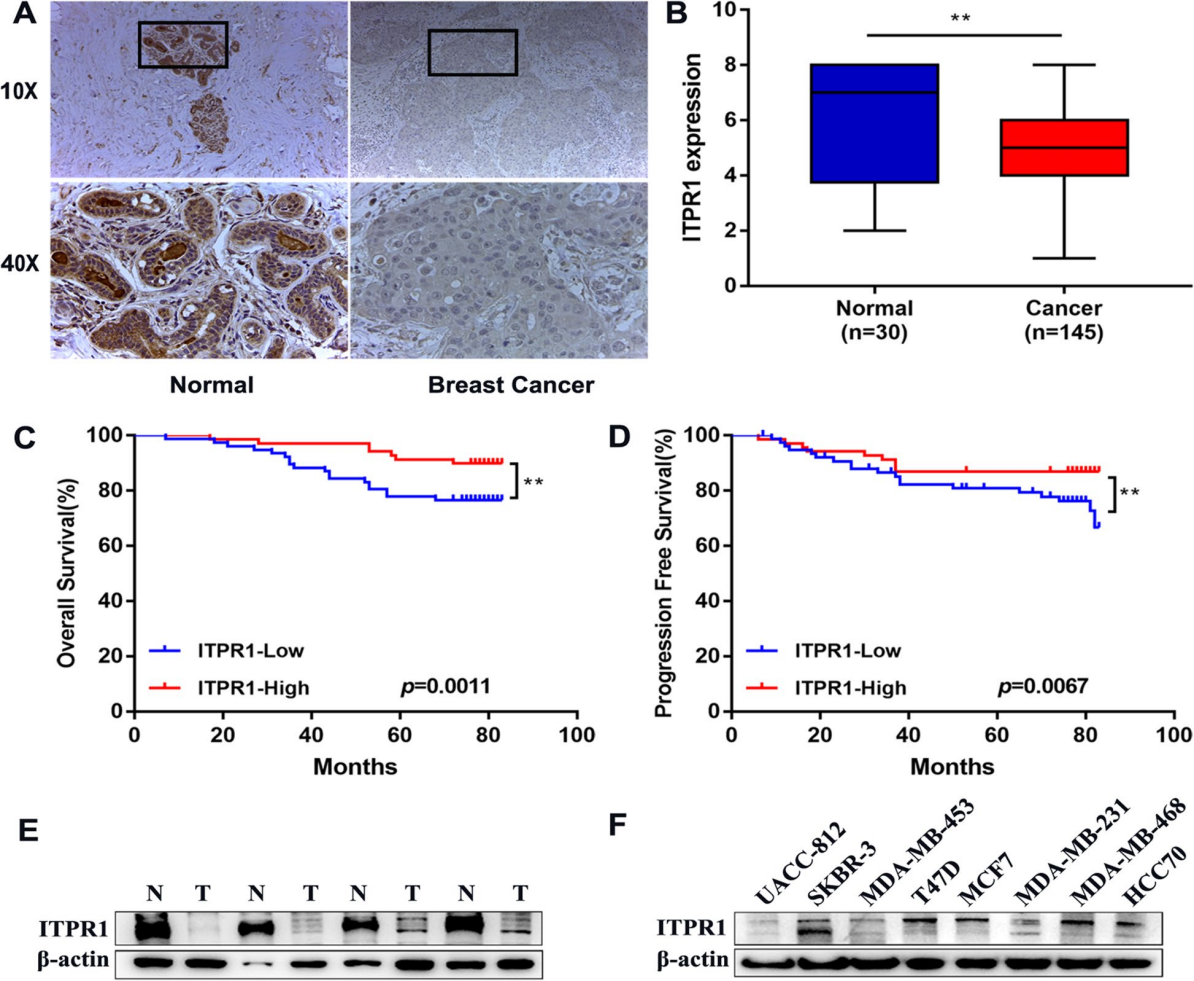
ITPR1 expression is decreased in breast cancer and correlates with prognosis. **A** Representative immunohistochemistry (IHC) images of ITPR1 in Breast Cancer tissues and normal tissues. **B** Box plots of normal and cancer expression of ITPR1 gene in Breast tissues. **C**-**D** Prognostic value of expression of ITPR1 in patients with breast cancer (**C**) Overall Survival (**D**) Progression Free Survival. (Set ITPR1 score < 6 as low expression, ITPR1 score ≥ 6 as high expression) (**E**) ITPR1 was measured in different breast tissues by Western blot. **F** ITPR1 was measured in different breast cells by Western blot

The analysis of 145 cases of breast cancer and 30 cases of adjacent normal tissues from the Harbin Medical University Cancer Center (HMUCC) further verified the low expression of ITPR1 in breast cancer (Fig. 5A, Show the comparison between normal breast tissue and HER2 positive tissue, Fig. 5B). At the same time, in order to explore the relationship between ITPR1 expression and the prognosis of breast cancer patients, we separately assessed the effects of ITPR1 expression and clinical results on overall survival and progression-free survival of breast cancer. The results showed that high expression of ITPR1 significantly prolonged the prognosis of patients than low ITPR1 (Fig. 5C, D). After that, we continued to explore the correlation between ITPR1 and the clinicopathological characteristics of breast cancer patients. ITPR1 was negatively correlated with tumor size, tumor lymph node metastasis (TNM) staging, lymph node metastasis (LNM), estrogen receptor (ER), progesterone receptor (PR) and HER2 status (*P* < 0.05). However, no significant associations were found between ITPR1 and age, Ki-67 and p53 status (Table 2). In univariate Cox regression models, Lymph Node Metastasis (*P* < 0.001), ITPR1 (*P* = 0.049), were found to achieve statistical significance (Table 3). Multivariate Cox proportional hazards model analysis showed that Lymph Node Metastasis (*P* < 0.001; HR, 24.845; 95%CI, 4.567–135.157), TNM stages (*P* = 0.037; HR, 0.152; 95% CI, 0.026–0.895) were the independent prognostic indicator of overall survival (Table 3). After that, we continued to use western blot to verify the expression of ITPR1 in breast cancer tissues and cells. The results showed that compared with normal tissues, the expression of ITPR1 in cancer tissues was significantly reduced. In cells, the expression of ITPR1 in the luminal type (T47D, MCF7) was relatively high, while the expression in HER2 + (UACC-812, SKBR-3 and MDA-MB-453) was relatively low (Fig. 5E, F).

**Table 2:** Association of ITPR1 level with clinical and pathological characteristics of breast cancer patients

variables	Tumor ITPR1 expression		*P* value
	ITPR1^Low^	ITPR1^High^	
Age (years)			0.563
≤ 50	36	36	
> 50	40	33	
Tumor size (cm)			
≤ 2	34	38	0.046*
> 2, ≤ 5	38	31	
> 5	4	0	
TNM stages			
I-II	15	33	< 0.001*
III-IV	61	36	
Lymoh Node Metastasis			0.001*
Negative	32	48	
Positive	44	21	
ER status			0.018*
Negative	59	41	
Positive	17	28	
PR status			0.001*
Negative	66	43	
Positive	10	26	
HER2 status			0.037*
Negative	40	48	
Positive	36	21	
P53 status			0.146
Negative	40	28	
Positive	36	41	
Ki-67 status			0.465
Negative	19	21	
Positive	57	48	

*Note*:A χ^2^ test was used for camparing gtoups between low and high ITPR1expression. *n* = 145, *p* < 0.05 was considered significant

**Table 3:** Univariate analysis and multivariate analysis of overall survival in patients with Breast Cancer (*n* = 145)

Variable	Univariate analysis			Multivariate analysis		
	*P* value	Hazard Ratio	95% confidence interval	*P* value	Hazard Ratio	95% confidence interval
Age	0.105	2.017	0.863–4.714			
Tumor size	0.368	1.386	0.681–2.820			
TNM stages	0.074	2.663	0.910–7.792			
Lymph Node Metastasis	** < 0.001**	7.106	2.427–20.810	**0.001**	6.336	2.122–18.914
ER status	0.495	0.725	0.288–1.827			
PR status	0.612	0.775	0.289–2.075			
HER2 status	0.114	1.910	0.855–4.263			
P53 status	0.218	0.601	0.267–1.353			
Ki-67 status	0.440	1.475	0.551–3.950			
ITPR1	**0.049**	0.413	0.171–0.997	0.291	0.617	0.252–1.513

### GO and KEGG enrichment analysis of ITPR1 and its 20 co-expression genes

**Fig. 6:**
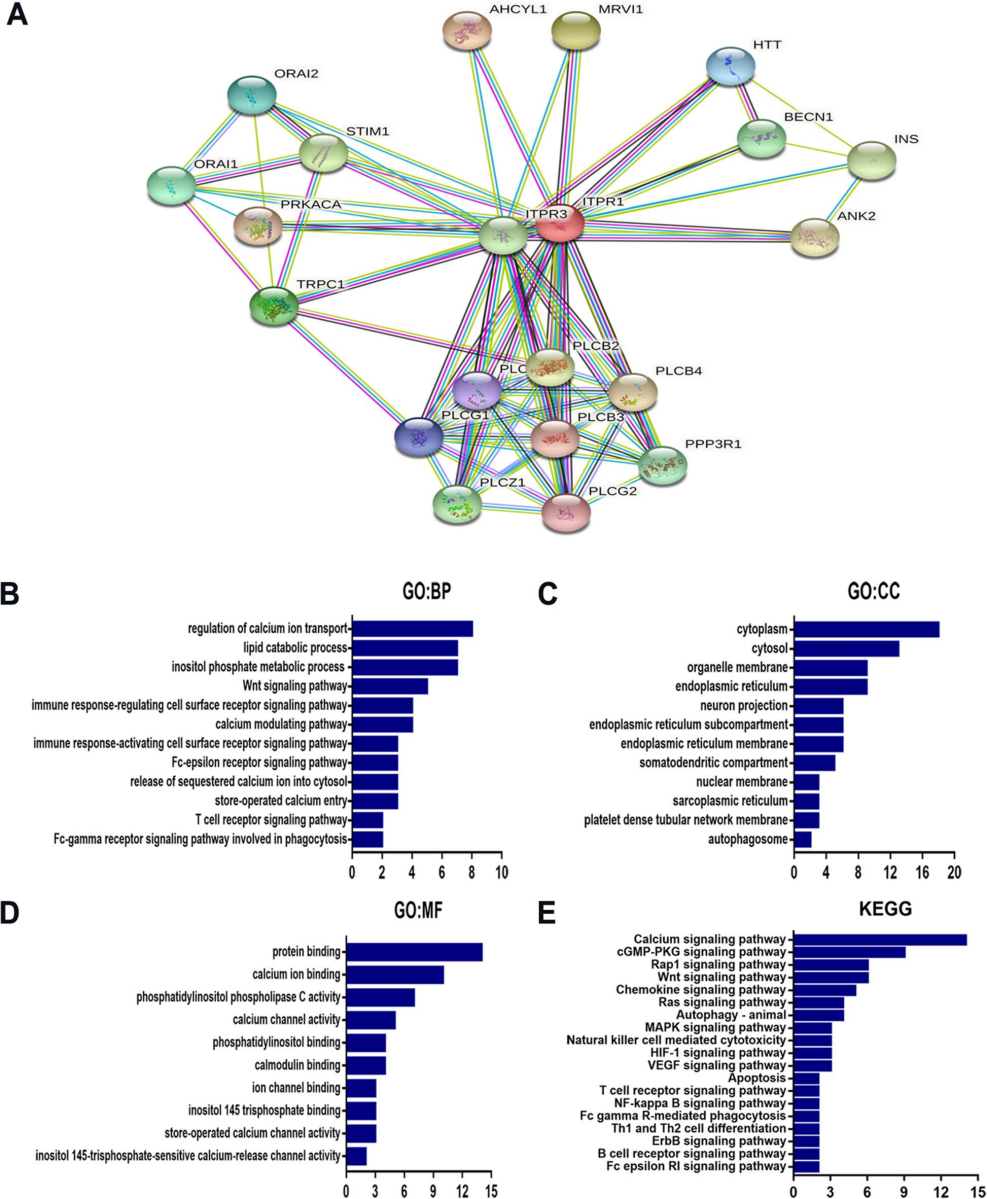
GO and KEGG enrichment analysis of ITPR1 and its 20 co-expression genes. (STRING). **A** PPI network. The nodes meant proteins; the edges meant the interaction of proteins (**B**) BP (**C**) CC (**D**) MF (**E**) KEGG

After analyzing the expression of ITPR1 and the prognostic value of breast cancer patients, we used STRING's "co-expression" module to analyze 20 co-expressed genes that were significantly related to ITPR1. Subsequently, we builted a comprehensive network through STRING. The results in Fig. 6A reveal that autophagosome-related genes, including BECN1 and calcium signaling pathway participant genes, such as STIM1, ORAI1, and ORAI2, were closely related to ITPR1. Predict the function of ITPR1 and its 20 co-expressed genes by analyzing the Annotation, Visualization, and Integrated Discovery database (STRING) of Gene Ontology (GO) and Kyoto Encyclopedia of Genes and Genomes (KEGG) [37]. As shown in Fig. 6B-E, we found that gene sets related to ITPR1 were enriched in functions related to these BP, CC and MF. Among them, BP such as GO: 0,051,924 (regulation of calcium ion transport), GO: 0,038,096 (Fc -γ receptor signaling pathway involved in phagocytosis), GO: 0,050,852 (T cell receptor signaling pathway), GO: 0,002,768 (immune response regulating cell surface receptor signaling pathway), GO: 0,016,055 (Wnt signaling pathway), GO: 0,007,223 (calcium regulation pathway) and GO: 0,043,647 (phosphoinositide metabolism process) (Fig. 6B). In addition, CC included GO: 0,031,095 (platelet dense tubular network membrane), GO: 0,016,529 (sarcoplasmic reticulum), GO: 0,005,776 (autophagosome) and GO: 0,098,827 (endoplasmic reticulum subcompartment), GO: 000,578 (internal Plasma reticulum membrane) GO:0,005,829 (cytosol) (Fig. 6C). MF, such as GO:0,070,679 (inositol 1,4,5 triphosphate binding), GO:0,044,325 (ion channel binding), GO:0,005,516 (calmodulin binding), GO:0,005,509 (calcium Ion binding) and GO:0,005,515 (p rotein binding) (Fig. 6D). KEGG analysis found 19 pathways related to the function of ITPR1 in breast adenocarcinoma. Among them, hsa04370 (VEGF signaling pathway), hsa04066 (HIF-1 signaling pathway), hsa04310 (Wnt signaling pathway), hsa04664 (Fc epsilon RI signaling pathway), hsa04662 (B cell receptor signaling pathway), hsa04658 (Th1 and Th2) Cell differentiation), hsa04660 (T cell receptor signaling pathway) and hsa04020 (calcium signaling pathway) were closely related to the function of ITPR1 (Fig. 6E).

### ITPR1 expression is correlated with immune infiltration level in breast cancer

**Fig. 7:**
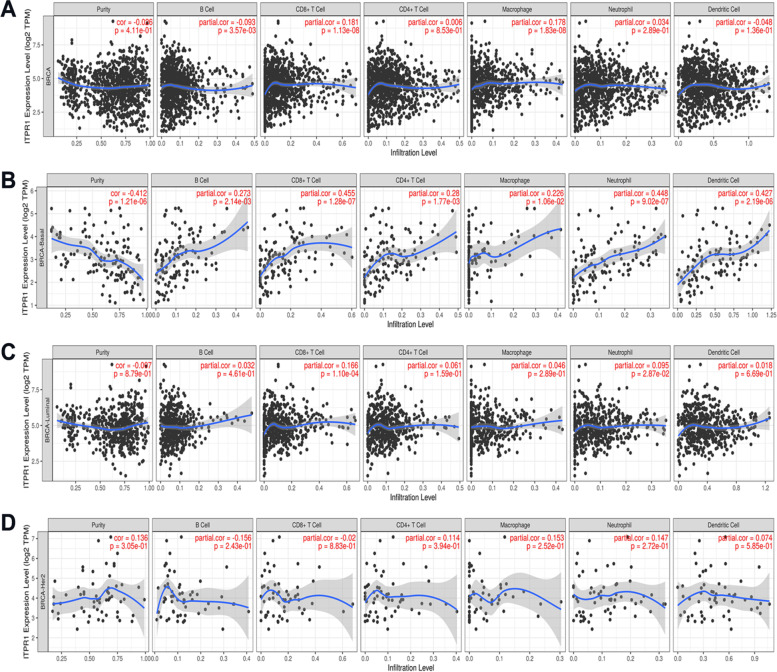
Correlation of ITPR1 expression with immune infiltration level in Breast Cancer (TIMER). **A** BRCA (**B**) BRCA-Basal (**C**) BRCA-Luminal and (**D**) BRCA-Her2

Through the analysis of KEGG, it is found that ITPR1 is widely involved in immune regulation pathways, such as: Fc epsilon RI signaling pathway, B cell receptor signaling pathway, Th1 and Th2 cell differentiation, T cell receptor signaling pathway. In oncology, immunotherapy is also a hot topic. Studies have shown that it has significant curative effects in kidney cancer, melanoma and non-small cell lung cancer [38–40]. Thus, we assessed the correlation between ITPR1 and the level of breast cancer immune invasion from the TIMER database. In order to understand the correlation between ITPR1 and different infiltrating immune cells including CD4 + T cells, CD8 + T cells, B cells, macrophages, neutrophils and DCs, TIMER was used to determine the relationship BRCA-Basal, BRCA-Luminal and BRCA-Her2 (Fig. 7). The results showed that high ITPR1 expression level had positive correlations with infiltrating levels of B cells (r = 0.273, *P* = 2.14e-03), CD8 + T cells (r = 0.455, *P* = 1.28e-07), CD4 + T cells (r = 0.28, *P* = 1.77e-03), macrophages (r = 0.226, *P* = 1.06e-02), dendritic cell (r = 0.448, *P* = 9.02e-07) and neutrophils (r = 0.427, *P* = 2.19e-06) in BRCA-Basal cancer (Fig. 7B). However, the expression of ITPR1 in BRCA-Lunimal and BRCA-Her2 has no significant correlation with most immune cells (Fig. 7C-D). These findings suggest that ITPR1 might play a specific role in immune infiltration of BRCA-Basal cancer.

### The correlation between ITPR1 and different immune biomarkers

After analyzing the correlation between ITPR1 and immune cells, we further studied the correlation between the biomarker genes in these immune cells or their subgroups and ITPR1, providing a basis for screening suitable therapeutic targets. TIMER analysis found that after the correlation adjustment of tumor purity, the results showed that ITPR1 in BRCA-Basal was closely related to the immune markers of most immune cells (Table 4). It is worth noting that the CD86 of monocyte, CD68 of TAM, VSIG4, MS4A4A of M2 macrophages, ITGAM of neutrophil, HLA-DRA, HLA-DPA1, ITGAX of dendritic cells, STAT4, STAT1 of Th1, CCR8, TGFB1 of Treg showed moderately correlate with ITPR1 expression (0.40 > COR value ≥ 0.30) (Table 4). CSF1R of monocytes, IL10 of TAM, NRP1 of dendritic cells, HAVCR2 of T cell exhaustion presented strong correlation with ITPR1 expression (COR value ≥ 0.40) (Table 4). Similarly, in BRCA-Lunimal and BRCA-Her2, the correlation between ITPR1 expression and immune cell-related markers has no obvious significance (Table 4).

**Table 4:** Correlation between ITPR1 and related gene markers of immune cells

Description	Gene markers	BRCA				BRCA-Basal				BRCA-Luminal				BRCA-Her2			
		**None**		**Purity**		**None**		**Purity**		**None**		**Purity**		**None**		**Purity**	
		**Cor**	***P***	**Cor**	***P***	**Cor**	***P***	**Cor**	***P***	**Cor**	***P***	**Cor**	***P***	**Cor**	***P***	**Cor**	**P**
T cell	CD3D	**-0.099**	**0.000**	**-0.13**	**0.000**	**0.405**	**0.000**	0.165	0.063	-0.043	0.291	-0.032	0.458	-0.128	0.301	-0.154	0.250
	CD3E	-0.058	0.055	**-0.08**	**0.011**	**0.416**	**0.000**	**0.178**	**0.045**	-0.034	0.399	-0.023	0.6	-0.146	0.239	-0.176	0.186
	CD2	-0.056	0.063	**-0.076**	**0.016**	**0.476**	**0.000**	**0.281**	**0.001**	-0.016	0.683	0.002	0.962	-0.151	0.223	-0.217	0.102
CD8 + T cell	CD8A	0.004	0.903	-0.012	0.699	**0.394**	**0.000**	**0.179**	**0.043**	0.012	0.773	0.031	0.475	-0.13	0.292	-0.165	0.216
	CD8B	**-0.102**	**0.000**	**-0.127**	**0.000**	**0.4**	**0.000**	**0.3**	**0.000**	0.01	0.807	0.007	0.869	-0.146	0.237	-0.171	0.201
B cell	CD19	**-0.122**	**0.000**	**-0.145**	**0.000**	**0.284**	**0.000**	-	0.99	-0.031	0.446	-0.023	0.587	-0.115	0.356	**-0.563**	**0.000**
	CD79A	**-0.089**	**0.000**	**-0.11**	**0.000**	**0.284**	**0.000**	-0.01	0.914	0.006	0.879	0.022	0.602	-0.123	0.319	-0.11	0.41
Monocyte	CD86	0.006	0.843	-0.001	0.976	**0.53**	**0.000**	**0.362**	**0.000**	0.012	0.757	0.027	0.525	0.084	0.5	0.073	0.587
	CD115(CSF1R)	**0.134**	**0.000**	**0.134**	**0.000**	**0.605**	**0.000**	**0.472**	**0.000**	**0.137**	**0.000**	**0.151**	**0.000**	0.097	0.432	0.129	0.336
TAM	CCL2	**-0.087**	**0.000**	**-0.084**	**0.000**	**0.4.3**	**0.000**	**0.261**	**0.003**	-0.042	0.293	-0.02	0.641	-0.051	0.681	-0.02	0.883
	CD68	-0.012	0.681	-0.023	0.466	**0.548**	**0.000**	**0.389**	**0.000**	-0.062	0.127	-0.061	0.158	0.145	0.242	0.16	0.230
	IL10	-0.003	0.918	-0.005	0.883	**0.538**	**0.000**	**0.402**	**0.000**	-0.02	0.619	-0.013	0.76	0.033	0.792	0.006	0.967
M1 Macrophage	IRF5	0.002	0.952	-0.018	0.571	**0.393**	**0.000**	**0.225**	**0.011**	0	0.997	-0.7	0.865	-0.014	0.911	-0.017	0.899
	INOS(NOS2)	-0.018	0.544	-0.013	0.693	0.034	0.689	0.088	0.325	0.03	0.45	0.008	0.853	0.157	0.204	0.151	0.257
	COX2(PTGS2)	0.007	0.806	0.015	0.636	0.128	0.13	**0.202**	**0.022**	**0.128**	**0.001**	**0.134**	**0.002**	0.185	0.134	0.156	0.242
M2 Macrophage	CD163	0.04	0.181	0.035	0.277	**0.589**	**0.000**	**0.46**	**0.000**	0.076	0.059	**0.088**	**0.041**	0.177	0.152	0.207	0.119
	VSIG4	**0.084**	**0.000**	**0.073**	**0.021**	**0.511**	**0.000**	**0.383**	**0.000**	**0.096**	**0.018**	**0.093**	**0.029**	0.155	0.211	0.179	0.179
	MS4A4A	0.048	0.11	0.041	0.199	**0.525**	**0.000**	**0.384**	**0.000**	0.032	0.428	0.041	0.34	-0.028	0.82	-0.036	0.787
Neutrophils	CD11b(ITGAM)	**0.088**	**0.000**	**0.083**	**0.000**	**0.53**	**0.000**	**0.387**	**0.000**	0.031	0.443	0.032	0.461	**0.261**	**0.033**	**0.316**	**0.019**
	CCR7	-0.012	0.69	-0.023	0.465	**0.401**	**0.000**	0.156	0.078	0.011	0.79	0.02	0.646	-0.005	0.965	-0.018	0.891
Natural killer cell	KIR2DL1	**-0.074**	**0.014**	**-0.093**	**0.000**	**0.188**	**0.026**	0.032	0.717	0.004	0.917	0.002	0.955	-0.077	0.538	-0.083	0.538
	KIR2DL3	**-0.061**	**0.043**	-0.059	0.065	**0.336**	**0.000**	**0.191**	**0.030**	-0.027	0.496	0.008	0.86	-0.066	0.595	-0.084	0.532
	KIR2DL4	**-0.138**	**0.000**	**-0.144**	**0.000**	**0.297**	**0.000**	0.121	0.173	-0.049	0.222	-0.037	0.386	-0.06	0.628	-0.073	0.584
	KIR3DL1	**-0.068**	**0.024**	**-0.078**	**0.013**	**0.335**	**0.000**	**0.19**	**0.032**	-0.006	0.876	0.002	0.962	-0.004	0.973	-0.034	0.799
	KIR3DL2	**-0.087**	**0.004**	**-0.096**	**0.002**	**0.31**	**0.000**	0.145	0.102	-0.031	0.446	0.001	0.978	-0.129	0.297	-0.206	0.121
	KIR3DL3	**-0.063**	**0.038**	**-0.072**	**0.022**	0.134	0.115	0.027	0.766	-0.013	0.741	0.009	0.83	-0.161	0.194	-0.166	0.212
	KIR2DS4	**-0.079**	**0.008**	**-0.093**	**0.003**	**0.184**	**0.029**	-0.003	0.977	-0.01	0.806	-0.007	0.878	-0.236	0.055	**-0.263**	**0.044**
Dendritic cell	HLA-DPB1	0.016	0.602	-0.003	0.936	**0.44**	**0.000**	**0.232**	**0.008**	0.025	0.533	0.027	0.528	-0.063	0.613	-0.1	0.455
	HLA-DQB1	0.016	0.602	**-0.071**	**0.026**	**0.387**	**0.000**	**0.211**	**0.017**	-0.019	0.634	-0.013	0.753	-0.088	0.476	-0.102	0.448
	HLA-DRA	0.047	0.118	0.041	0.197	**0.498**	**0.000**	**0.326**	**0.000**	0.039	0.33	0.052	0.228	-0.044	0.722	-0.071	0.596
	HLA-DPA1	**0.108**	**0.000**	**0.114**	**0.000**	**0.502**	**0.000**	**0.343**	**0.000**	**0.08**	**0.048**	**0.097**	**0.024**	-0.093	0.454	-0.133	0.319
	BDCA-1(CD1C)	**0.125**	**0.000**	**0.141**	**0.000**	**0.347**	**0.000**	0.169	0.056	**0.125**	**0.002**	**0.146**	**0.000**	0.07	0.572	0.085	0.524
	BDCA-4(NRP1)	**0.216**	**0.000**	**0.213**	**0.000**	**0.597**	**0.000**	**0.524**	**0.000**	0.066	0.103	0.055	0.203	-0.019	0.878	0.004	0.979
	CD11c(ITGAX)	-0.005	0.863	-0.011	0.722	**0.515**	**0.000**	**0.338**	**0.000**	-0.044	0.277	-0.04	0.352	0.055	0.656	0.099	0.461
Helper T cell 1 (TH1)	T -bet (TBX21)	**-0.069**	**0.022**	**-0.095**	**0.003**	**0.441**	**0.000**	**0.247**	**0.005**	-0.002	0.958	0.017	0.691	0.076	0.539	-0.117	0.382
	STAT4	0.032	0.293	0.018	0.573	**0.548**	**0.000**	**0.388**	**0.000**	0.038	0.351	0.041	0.334	-0.191	0.122	-0.223	0.092
	STAT1	**0.068**	**0.024**	**0.061**	**0.054**	**0.501**	**0.000**	**0.388**	**0.000**	0.03	0.451	0.036	0.398	0.077	0.537	0.007	0.96
	IFNG	**-0.14**	**0.000**	**-0.162**	**0.000**	**0.359**	**0.000**	**0.186**	**0.036**	-0.032	0.423	-0.016	0.705	-0/019	0.88	-0.034	0.798
	TNF	**-0.116**	**0.000**	**-0.097**	**0.002**	**0.278**	**0.000**	0.167	0.060	0.012	0.773	0.047	0.275	0.019	0.876	0.058	0.663
Regulatory cell (Treg)	FOXP3	**-0.128**	**0.000**	**-0.136**	**0.000**	**0.394**	**0.000**	**0.175**	**0.048**	**-0.11**	**0.006**	**-0.101**	**0.019**	-0.12	0.331	-0.176	0.187
	CCR8	0.021	0.489	0.022	0.489	**0.524**	**0.000**	**0.364**	**0.000**	-0.011	0.776	0.002	0.959	**0.277**	**0.024**	**0.279**	**0.034**
	TGFB1 (TGFβ)	**0.119**	**0.000**	**0.117**	**0.000**	**0.513**	**0.000**	**0.348**	**0.000**	-0.075	0.061	**-0.088**	**0.041**	0.153	0.214	0.161	0.228
T cell exhaustion	PD-1 (PDCD1)	**-0.142**	**0.000**	**-0.171**	**0.000**	**0.319**	**0.000**	0.087	0.326	-0.054	0.179	-0.04	0.35	-0.098	0.43	-0.093	0.486
	CTLA4	**-0.179**	**0.000**	**-0.199**	**0.000**	**0.428**	**0.000**	**0.208**	**0.019**	-0.069	0.086	-0.046	0.282	-0.028	0.819	-0.06	0.654
	LAG3	**-0.241**	**0.000**	**-0.248**	**0.000**	**0.341**	**0.000**	0.148	0.095	**-0.096**	**0.017**	-0.068	0.114	-0.032	0.799	-0.07	0.602
	TIM-3 (HAVCR2)	0.047	0.12	0.035	0.264	**0.575**	**0.000**	**0.426**	**0.000**	-0.015	0.716	-0.015	0.724	0.128	0.302	0.131	0.329
	GZMB	**-0.243**	**0.000**	**-0.279**	**0.000**	**0.349**	**0.000**	0.144	0.105	**-0.09**	**0.026**	**-0.085**	**0.048**	-0.093	0.452	-0.111	0.407

### Co-expression analysis of ITPR1 gene

**Fig. 8:**
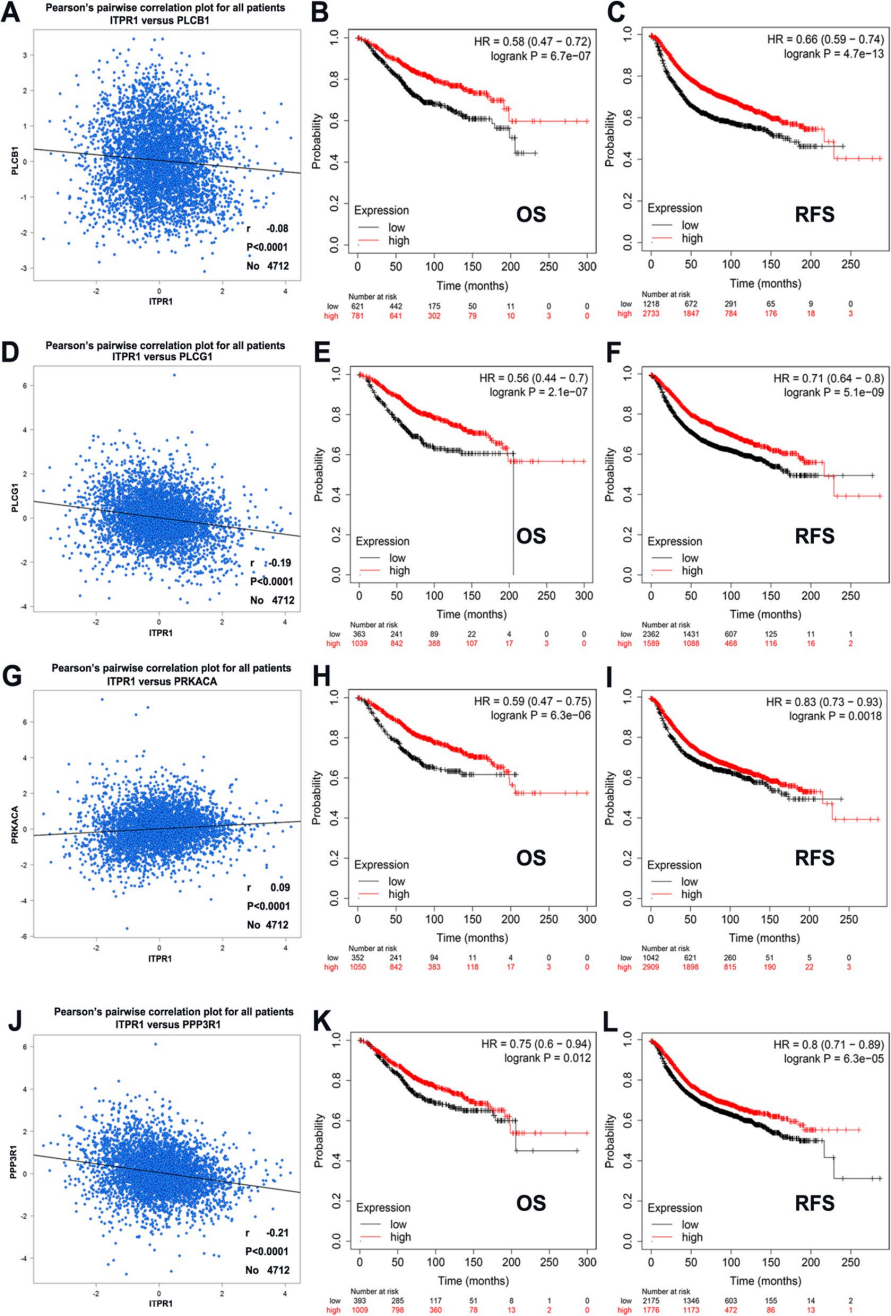
Co-expression analysis of gene ITPR1 and other genes. ( bc-GenExMiner software and Kaplan–Meier plotter). **A**,** D, G, J** The correlation between ITPR1 and co-expressed genes. **B**-**C**, **E**–**F**, **H**-**I**,**K**-**L** Survival analysis of ITPR1 and co-expressed genes

In addition to immune pathways, we also found that ITPR1 can regulate VEGF signaling pathway, HIF-1 signaling pathway and Calcium signaling pathway, and these pathways themselves also regulate each other (Supplementary Fig. 3). Based on the 20 co-expression gene analyzed in the STRING database, four genes involved in these four pathways were found, namely PLCB1, PLCG1, PRKACA, and PPP3R1 (Supplementary Table 4). We found the co-expression gene PRKACA (Fig. 8G, *P* < 0.0001, *r* = 0.09) was associated with ITPR1 positively and PLCB1 (Fig. 8A, *P* < 0.0001, *r* = -0.08), PLCG1 (Fig. 8D, *P* < 0.0001, *r* = -0.19), PPP3R1 (Fig. 8J, *P *< 0.0001, *r *= -0.21) were associated with ITPR1 negatively according to Bc-GenExMiner v4.3. However, we found that ITPR1 is correlated with PLCG1 and PPP3R1, while the correlation between PLCB1 and PRKACA is lower. Furthermore, we used the Kaplan–Meier plotter to analyze the prognostic value of ITPR1 and co-expressed genes in patients with breast cancer. As shown in the figure, predicting that breast cancer patients with high expression of ITPR1 combined with co-expressed genes will have high OS and RFS, which was more meaningful than analyzing the prognosis of co-expressed genes alone (Fig. 8B-C, E–F, H-I, K-L, Supplementary Fig. 4). These further proved that ITPR1 could improve the prognosis of breast cancer patients.

## Discussion

In 2020, female breast cancer has now surpassed lung cancer, becoming the main cause of global cancer incidence. It is the fifth leading cause of cancer deaths worldwide. Among women, breast cancer accounts for one-quarter of cancer cases and one-sixth of cancer deaths. It ranks first among most countries (159 out of 185 countries) in morbidity and mortality in 110 countries [2]. Therefore, it is necessary to find new targets for breast cancer treatment.

Many biological functions are regulated by intracellular calcium ions, including cell proliferation, gene transfer, and cell death [9, 41–44]. Calcium signals are usually initiated by the combination of hormones or growth factors to produce a signal cascade of diacylglycerol and inositol 1,4,5-triphosphate (IP3). Subsequently, IP3 binds to a specific receptor (ITPR) on the endoplasmic reticulum membrane, triggering the release of Ca^2+ ^[44].

ITPR consists of approximately 2700 amino acids [45, 46], including N-terminal domain (IP3 domain), C-terminal domain and regulatory domain. ITPR is an intrinsic membrane protein with 6 transmembrane segments [47, 48]. ITPR regulates receptor activity through post-translational modification (phosphorylation, ubiquitination, etc.) or interaction with regulatory proteins (such as chromogranin A and B, neuronal calcium receptor 1, etc.) [49–52].

There are three subtypes of ITPR: ITPR1, ITPR2 and ITPR3 [53, 54]. Among them, the research of ITPR1 is relatively extensive. ITPR1 releases Ca^2+^ at lower Ca^2+^ concentrations, and inhibits the channel in the opposite case [55, 56]. Studies have shown that ITPR1 is located in the endoplasmic reticulum, cytoplasm and close to the nucleus in hepatocytes and bile duct cells, and ITPR1 also plays an important role in the metabolism, proliferation and secretion of bile duct cells in liver cancer cells [57–60]. ITPR1 is down-regulated in head and neck tumors and esophageal cancer [23, 24]. However, there is no association analysis in breast cancer.

In our study, according to a variety of database studies, ITPR1 had lower expression in many tumors. The expression of ITPR1 in breast cancer tissue was down-regulated compared with normal tissue. The expression of ITPR1 was related to the clinicopathological characteristics of breast cancer.

At present, breast cancer is divided into carcinoma in situ and invasive carcinoma according to the scope of invasion. Different breast cancer cells may have differences in the expression of estrogen receptor, progesterone receptor and HER2 protein. According to these characteristics, breast cancer can be divided into hormone receptor-positive breast cancer, HER-positive breast cancer and triple-negative breast cancer. According to reports, SBR grade and NPI are prognostic factors for breast cancer. SBR is graded according to three indicators: whether there are duct formation, nucleus morphology, and mitotic figures. NPI scores based on tumor size, number of axillary lymph node metastases, and histological grade under microscope. The higher the SBR grade or the higher the NPI score, the worse the prognosis. The expression of ITPR1 in SBR grade and NPI gradually decreases, and the expression of ER positive, HER2 negative, lymph node negative, non-triple negative and non-basal-like states was significantly increased, and it was related to the prognosis of breast cancer. Therefore, the expression of ITPR1 gene might be a new biomarker factor affecting the prognosis of breast cancer.

According to the survival curve of different datasets of ITPR1 expression, the prognostic value of breast cancer was analyzed with PrognoScan database. Through the meta-analysis of survival curve data, 9 data sets with clinical statistical value were proposed. Breast cancer patients with elevated expression of ITPR1 had better overall survival, recurrence-free survival, disease-specific survival, and survival without distant metastasis. The same trend was confirmed in Kaplan–Meier Plotter. Next, continue to analyze the correlation between the expression level of ITPR1 and clinical variables, and found that the expression of ITPR1 was related to hormone receptor-positive breast cancer, HER-positive breast cancer and triple-negative breast cancer. Then, we used immunohistochemistry and western blot methods to verify the expression and prognosis of ITPR1 once again. Unfortunately, the *P*-values of ITPR1 were not always meaningful in the multivariate analysis.We suggest that small sample sizes may lead to nonsignificant *P*-values.In the future, we will increase the tumor samples to elucidate the relationship between ITPR1 and the expression and survival of various clinical variables.

Then, we discussed the predictive value of ITPR1 expression level for clinical treatment of breast cancer. The result show that the expression level of ITPR1 in chemotherapy patients was higher than that in non-chemotherapeutic patients. In bladder cancer, the overexpression of ITPR1 in drug-resistant cells could induce cell apoptosis and increase sensitivity to cisplatin [16]. So we suspect that high expression of ITPR1 can increase the sensitivity of breast cancer chemotherapy. According to the results the expression level of ITPR1 could predict the effect of chemotherapy, the AUC value was 0.589, *P* < 0.05. AUC (Area Under Curve) is defined as the area under the ROC curve and the coordinate axis. The value range of AUC is between 0.5 -1. The closer the AUC is to 1.0, the higher the authenticity of the detection method; when it is equal to 0.5, the authenticity is the lowest and it has no application value. According to the predicted AUC, although it is greater than 0.5, it is not significant. According to the results of bioinformatics analysis and prediction, we need further experimental verification to draw conclusions. It can be used as a candidate resource and early foundation for future scientific team experimental exploration. The above results suggested that the high expression of ITPR1 may affect the prognosis of breast cancer.

In order to better understand ITPR1, we defined for 20 proteins that interact with ITPR1 in the STRING database, and performed GO enrichment and KEGG pathway analysis. ITPR1 was involved in a variety of biological processes, molecular functions and cellular components, including regulation of calcium ion transport, Fc-gamma receptor signaling pathway involved in phagocytosis, T cell receptor signaling pathway, immune response-regulating cell surface receptor signaling pathway, autophagosome, Endoplasmic reticulum membrane, calcium ion binding. The related pathways mainly included: VEGF signaling pathway, HIF-1 signaling pathway, Wnt signaling pathway, Fc epsilon RI signaling pathway, B cell receptor signaling pathway, Th1 and Th2 cell differentiation and T cell receptor signaling pathway. It has been reported that the combination of TMEM173 and ITPR1 could control the release of calcium from the endoplasmic reticulum of macrophages and monocytes [15]. In kidney cancer, HIF2α affected the expression of ITPR1 and activated autophagy of target cells through NK-derived signals, thereby regulating NK-mediated killing [21]. Our analysis also showed that the expression of ITPR1 was related to immune infiltration, and the biological function of ITPR1 in breast cancer was unclear.

Immunotherapy is a very popular treatment method for all malignant tumors including breast cancer in recent years. Different from the previous biological treatment, it is a treatment method to control the tumor by using appropriate methods to adjust or enhance the body's immune response to tumor cells. Some of them can achieve surprising effects. Usually chemotherapy, endocrine therapy, and targeted therapy only target tumor cells. Immunotherapy is a way to truly kill tumors by strengthening one's own immune system, so immunotherapy is expected to become a new innovation in the field of tumor treatment following surgery, radiotherapy, chemotherapy, and endocrine therapy. Next, our research provided a new perspective on the potential function of ITPR1 in breast cancer immunology and its application as a cancer biomarker. In this study, it was found that the expression of ITPR1 was related to the multiple levels of immune infiltration in triple-negative breast cancer, but is not related to luminal and HER2 breast cancer. The results showed that CD8 + T cells (*r *= 0.455, *P* = 1.28e-05), CD4 + T cells (*r* = 0.28, *P* = 1.77e-03), and neutrophils (*r* = 0.226, *P* = 1.06e-02), dendritic cells (*r* = 0.448, *P* = 9.02e-07) and macrophages (*r* = 0.427, *P* = 2.19e-06) infiltration levels were in the middle to ITPR1 expression strong positive correlation. And the correlation between the expression of ITPR1 and immune cell marker genes also suggests that ITPR1 might play a role in the immune regulation of triple-negative breast cancer. Similarly, the correlation between luminal and HER2 breast cancer were weak. The results showed that ITPR1 is related to monocytes, M2 macrophages, TAM, Th1 cells, neutrophils and dendritic cells of triple-negative breast cancer. However, whether it can play a regulatory role in triple-negative breast cancer requires further exploration. According to the results of clinical studies, atezolizumab combined with chemotherapy is the first-line treatment for patients with unresectable locally advanced or metastatic PD-L1-positive triple-negative breast cancer. Atezolizumab has also become the first approved for use Immunotherapy drugs for breast cancer. And immunotherapy combined with chemotherapy is also the first-line treatment for triple-negative breast cancer. Combined with our analysis, ITPR1 is correlated with the level of immune infiltration in triple-negative breast cancer, and is highly expressed in chemotherapy. It suggests that the increase of ITPR1 may improve the sensitivity of immunotherapy combined with chemotherapy in the treatment of triple-negative breast cancer. Next, four proteins that participate in the VEGF signaling pathway, HIF-1 signaling pathway, immune signaling pathway and Calcium signaling pathway are identified: PLCB1, PLCG1, PRKACA, and PPP3R1. The correlation was verified again in BC-GenExMiner v4.3. And analyze the prognostic value of ITPR1 and related gene complexes. However, we found that ITPR1 is correlated with PLCG1 and PPP3R1, while the correlation between PLCB1 and PRKACA is lower. These correlations might suggest the possible mechanism of ITPR1 regulating breast cancer immune cell function, and provided preliminary guidance for the selection of therapeutic targets.

It should be noted that our results are based on the analysis of big data from various databases, and we have collected these data as comprehensively as possible. However, this discovery can only provide a preliminary theoretical basis and needs to be further verified by follow-up research and clinical trials.

## Conclusions

Compared with normal breast tissue, the expression of ITPR1 in breast cancer is lower. The higher expression of ITPR1 suggested favorable prognosis for patients. ITPR1 was related to the level of immune infiltration, especially in BRCA-Basal patients. All research results indicated that ITPR1 might affect breast cancer prognosis and participate in immune regulation.

## Supplementary Information

**Additional file 1.**

**Additional file 2.**

## Data Availability

All data are available via the corresponding author.
