# Supplementary material for: Systematic analysis of the expression and prognostic value of ITPR1 and correlation with tumor infiltrating immune cells in breast cancer
Source: BMC Cancer. 2022 Mar 21;22:297. doi: 10.1186/s12885-022-09410-w (PMC8939201; doi:10.1186/s12885-022-09410-w)
Supplement: Supplementary file 1 — Additional file 1. [file 12885_2022_9410_MOESM1_ESM.docx]

**Supplementary Information**

**ITPR1 serves as a Potential Prognostic Biomarker and is Associates With Immune Infiltration in Breast Cancer**

**Bing Han et al.**

**Supplementary Table 1.The Significant Changes of ITPR1 Expression in Transcription Level between Different Types of Breast Cancer and Normal Breast Tissues (Oncomine)**

| **Breast cancer subtype vs. Normal** | **P value** | **T test** | **Fold Change** | **Source and/or Reference** |
| --- | --- | --- | --- | --- |
| Ductal breast carcinoma in situ stroma | 6.40E-4 | -3.765 | -2.030 | Ma breast statistics |
| Invasive ductal breast carcinoma stroma | 0.003 | -3.289 | -2.950 | Ma breast statistics |
| Ductal breast carcinoma | 1.05E-7 | -8.099 | -4.478 | Richardson breast statistics |
| Medullary breast carcinoma | 1.64E-11 | -9.127 | -2.649 | Curtis breast statistics |
| Invasive breast carcinoma | 8.45E-12 | -7.354 | -2.454 | TCGA |
| Invasive ductal breast carcinoma | 2.54E-19 | -11.022 | -2.707 | TCGA |

**Supplementary Table 2. ITPR1 gene expression analysis in different clinical parameters of breast cancer with Bc-GenExMiner v4.3.**

| **Variables** | **No.of patients** | **ITPR1 mRNA** | **P value*** |
| --- | --- | --- | --- |
| **Nodal status** |  |  | **P=0.0003** |
| Negative | 2415 | Increase |  |
| Positive | 1646 | - |  |
| **ER** |  |  | **<0.0001** |
| Negative | 551 | - |  |
| Positive | 3911 | Increase |  |
| **PR** |  |  | **<0.0001** |
| Negative | 828 | - |  |
| Positive | 3498 | Increase |  |
| **HER-2** |  |  | **<0.0001** |
| Negative | 3582 | Increase |  |
| Positive | 661 | - |  |
| **Triple-negative status** |  |  | **<0.0001** |
| Non-triple-negative | 317 | Increase |  |
| Triple-negative | 4119 | - |  |
| **Basal-like status** |  |  | **<0.0001** |
| Non-basal-like | 3837 | Increase |  |
| Basal-like | 832 | - |  |
| **Basal-like and Triple-negative status** |  |  | **<0.0001** |
| Non-basal-like and Non-triple-negative | 3690 | Increase |  |
| Basal-like and Triple-negative | 267 | - |  |

* Statistical significance was determined by the Welch’s test.

**Supplementary Table 3. Different datasets to analyze the prognosis of ITPR1 gene expression in breast cancer**

| **Dataset** | **Probe ID** | **Endpoint** | **No.** | **Cox P-value** | **HR** |
| --- | --- | --- | --- | --- | --- |
| GSE3143 | Overall Survival | 32778_at | 158 | 0.015994 | 0.55 [0.34-0.89] |
| GSE11121 | Distant Metastasis Free Survival | 216944_s_at | 200 | 0.022286 | 0.64 [0.44-0.94] |
| GSE2034 | Distant Metastasis Free Survival | 216944_s_at | 286 | 0.003892 | 0.75 [0.61-0.91] |
| GSE2034 | Distant Metastasis Free Survival | 211323_s_at | 286 | 0.002011 | 0.80 [0.70-0.92] |
| GSE1456-GPL96 | Relapse Free Survival | 216944_s_at | 159 | 0.001938 | 0.54 [0.36-0.80] |
| GSE1456-GPL96 | Relapse Free Survival | 203710_at | 159 | 0.000277 | 0.47 [0.31-0.71] |
| GSE1456-GPL96 | Disease Specific Survival | 203710_at | 159 | 0.002136 | 0.47 [0.29-0.76] |
| GSE1456-GPL96 | Overall Survival | 203710_at | 159 | 0.046955 | 0.66 [0.43-0.99] |
| GSE1456-GPL97 | Relapse Free Survival | 240052_at | 159 | 0.012958 | 0.63 [0.44-0.91] |

**Supplementary Table 4. Genes Contained in the KEGG Pathway.**

| **KEGG** | **Genes** |
| --- | --- |
| Fc epsilon RI signaling pathway | PLCG1,PLCG2 |
| B cell receptor signaling pathway | PPP3R1,PLCG2 |
| ErbB signaling pathway | PLCG1,PLCG2 |
| Th1 and Th2 cell differentiation | PPP3R1,PLCG1 |
| Fc gamma R-mediated phagocytosis | PLCG1,PLCG2 |
| NF-kappa B signaling pathway | PLCG1,PLCG2 |
| T cell receptor signaling pathway | PPP3R1,PLCG1 |
| Apoptosis | ITPR1,ITPR3 |
| VEGF signaling pathway | PPP3R1,PLCG1,PLCG2 |
| HIF-1 signaling pathway | PLCG1,INS,PLCG2 |
| Natural killer cell mediated cytotoxicity | PPP3R1,PLCG1,PLCG2 |
| MAPK signaling pathway | PPP3R1,PRKACA,INS |
| Autophagy - animal | ITPR1,PRKACA,BECN1,INS |
| Ras signaling pathway | PLCG1,PRKACA,INS,PLCG2 |
| Chemokine signaling pathway | PLCB2,PRKACA,PLCB1,PLCB4,PLCB3 |
| Wnt signaling pathway | PPP3R1,PLCB2,PRKACA,PLCB1,PLCB4,PLCB3 |
| Rap1 signaling pathway | PLCG1,PLCB2,PLCB1,PLCB4,INS,PLCB3 |
| cGMP-PKG signaling pathway | PPP3R1,PLCB2,ITPR1,PLCB1,ITPR3,PLCB4,INS,MRVI1,PLCB3 |
| Calcium signaling pathway | PPP3R1,PLCG1,PLCB2,PLCZ1,ITPR1,PRKACA,PLCB1,ORAI2,ITPR3,PLCB4,PLCB3,STIM1,ORAI1,PLCG2 |


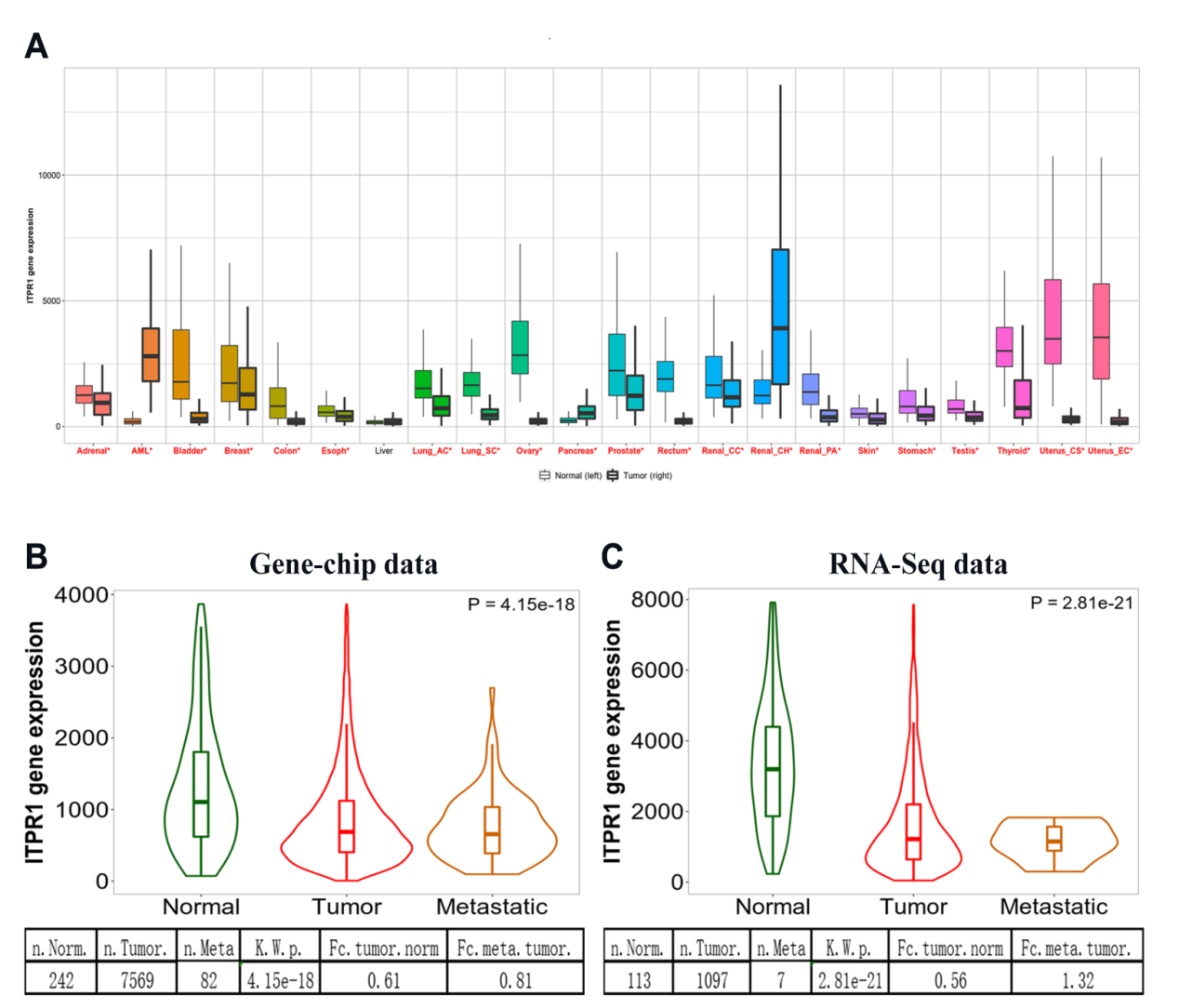


**Figure S1:** The expression of ITPR1 in distinct types of cancer diseases. **(A)** ITPR1 was abnormally expressed in pan cancers by TNMplot. Significant differences by Mann-Whitney U test are marked with red color. **(B)** Gene chip data of ITPR1 expression in normal, tumor, metastatic tissues. **(C)** RNA-seq data of ITPR1 expression in normal, tumor, metastatic tissues.


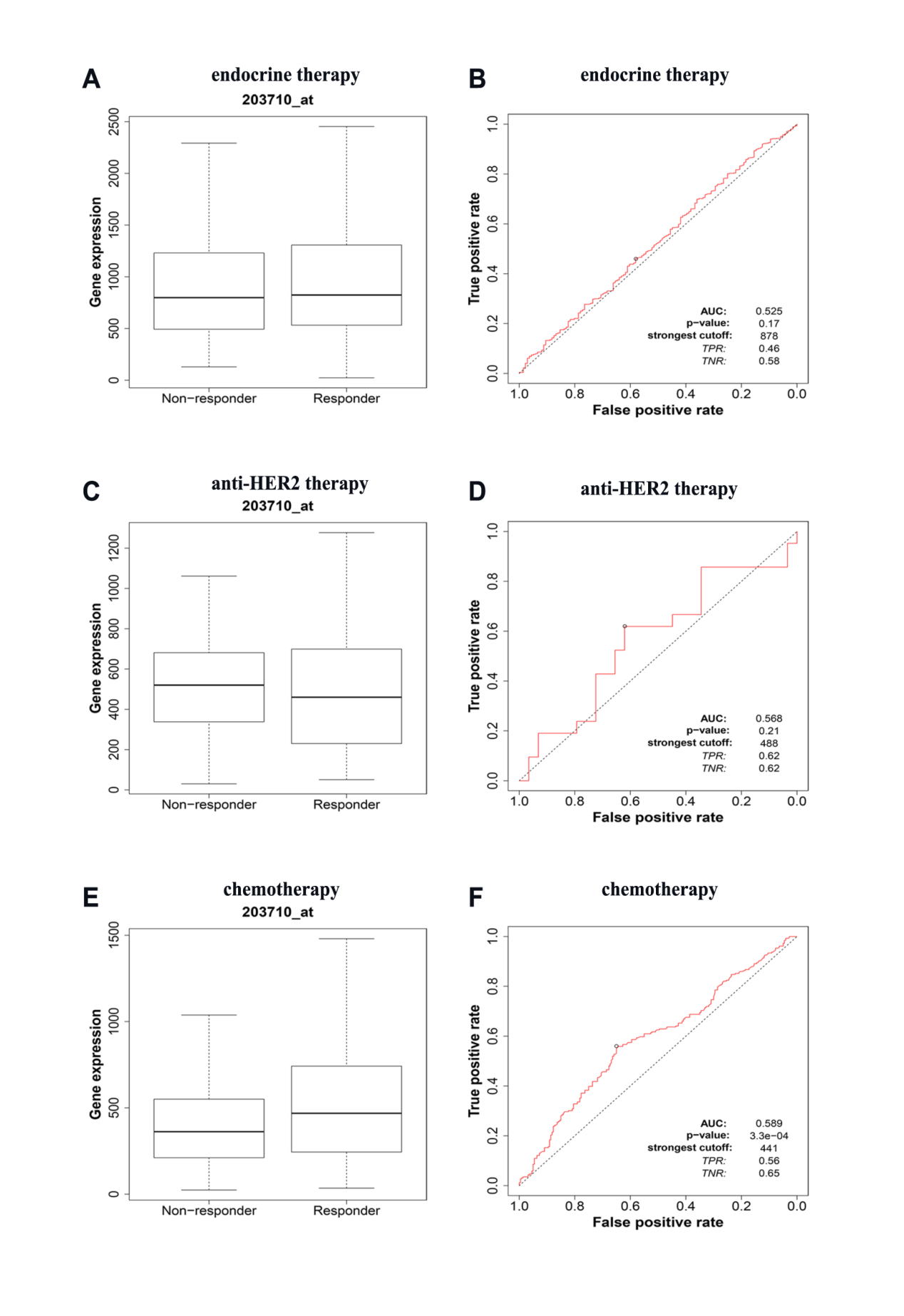


**Fig. S2:** The value of ITPR1 expression in the clinical treatment of breast cancer. **(A、C、E)** UBE2W expression between responders and non-responders in endocrine therapy、anti-HER2 therapy and chemotherapy of BRCA. **(B、D、F)** The ROC plot of UBE2W expression in predicting the effect of endocrine therapy、anti-HER2 therapy and chemotherapy in BRCA.


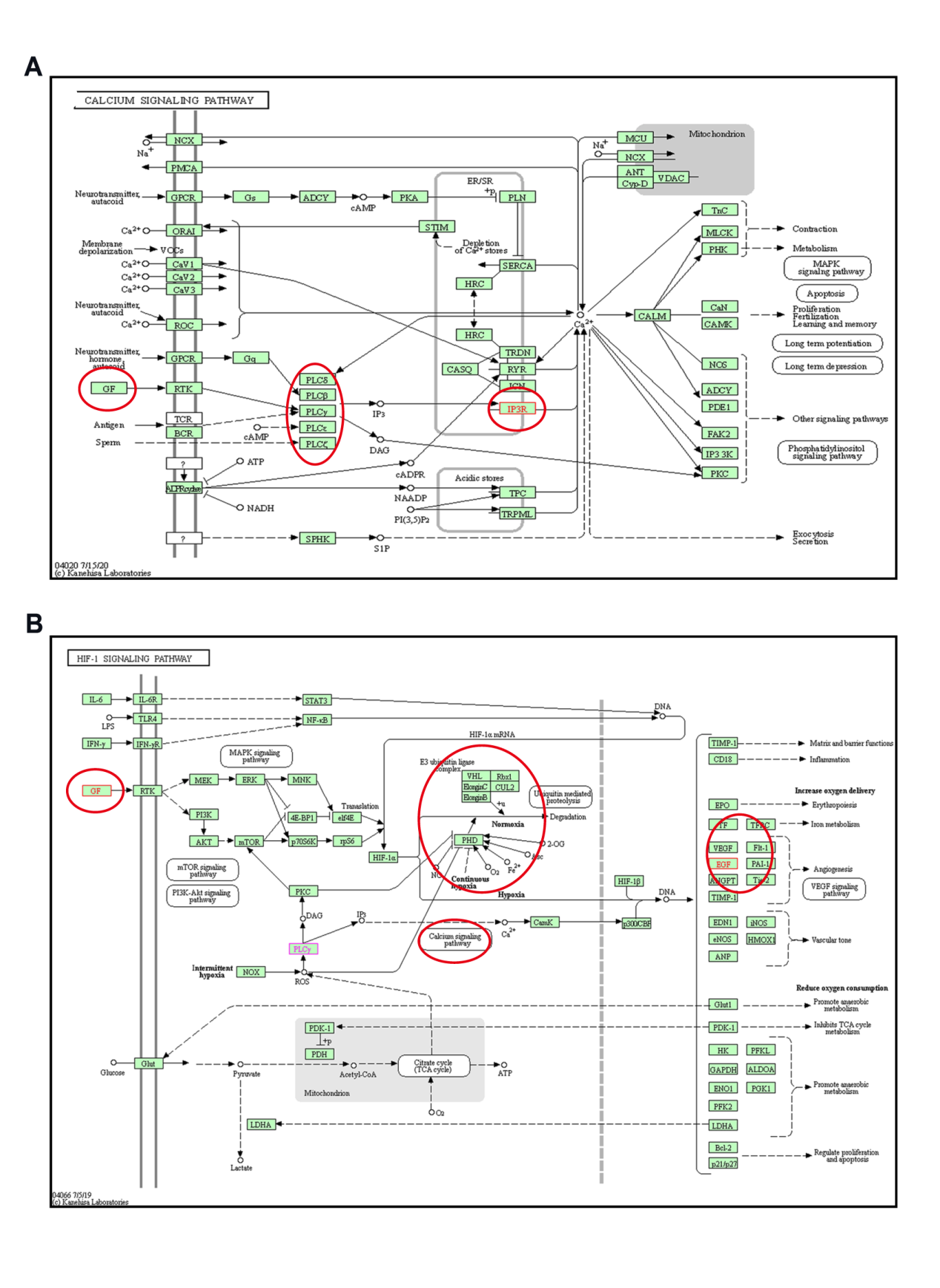


**Fig. S3:** Calcium Signaling Pathway and HIF-1 Signaling Pathway Regulated by the ITPR1. (KEGG) **(A)** calcium signaling pathway and **(B)** HIF-1 signaling pathway.


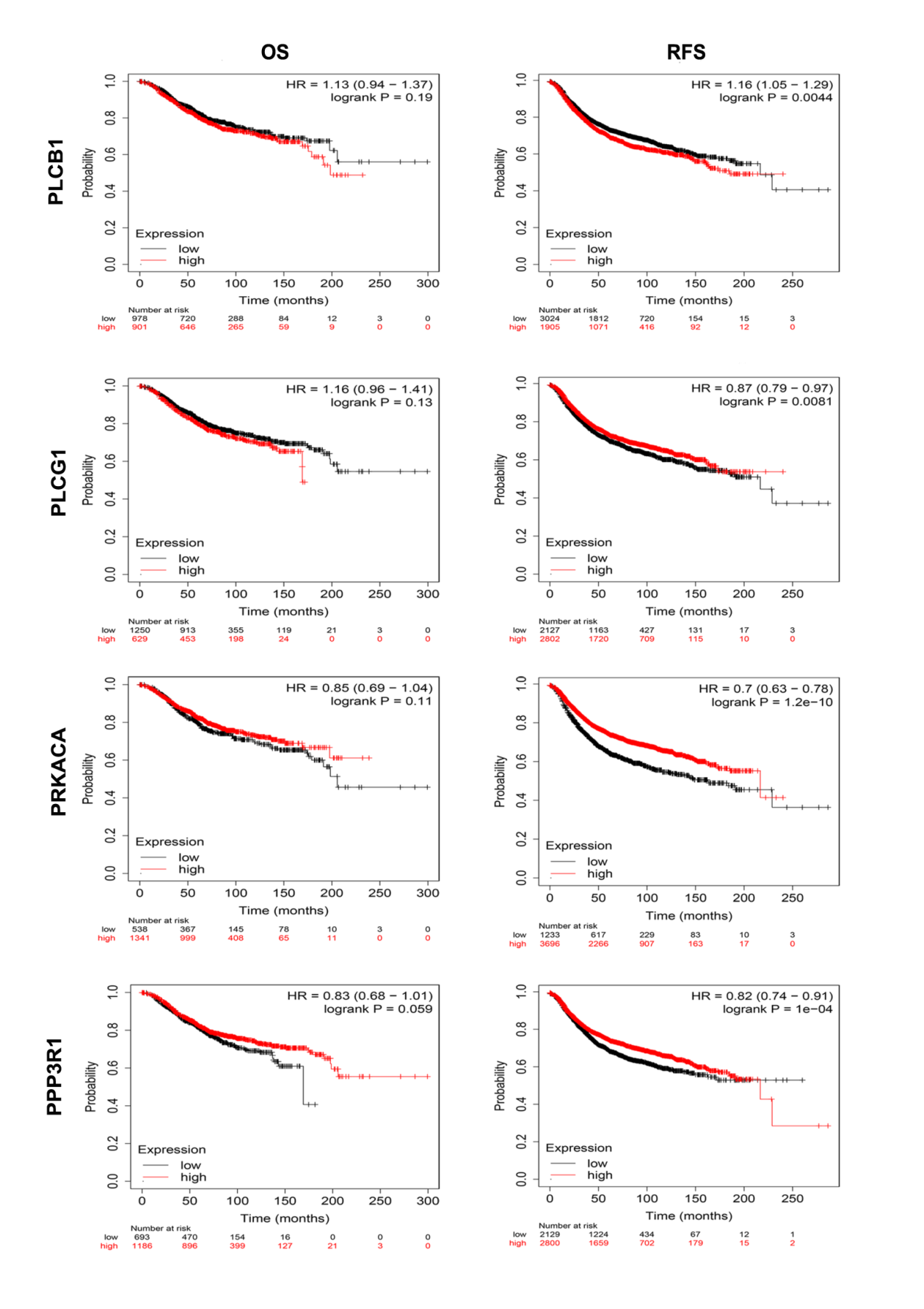


**Fig. S4:** Prognosis of genes related to ITPR1 (OS and RFS).
