# Supplementary material for: Systematic analysis of the expression and prognostic value of ITPR1 and correlation with tumor infiltrating immune cells in breast cancer
Source: BMC Cancer. 2022 Mar 21;22:297. doi: 10.1186/s12885-022-09410-w (PMC8939201; doi:10.1186/s12885-022-09410-w)

Fig5E ITPR1 was measured in different breast tissues by Western blot.


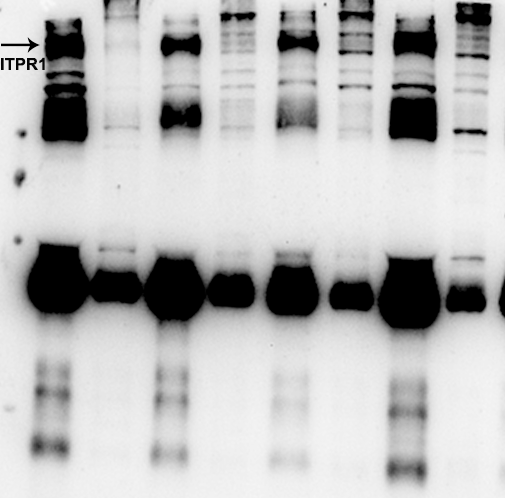

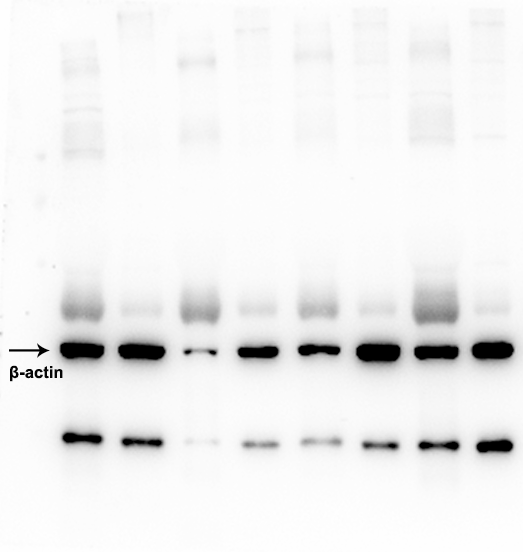


Fig5F ITPR1 was measured in different breast cells by Western blot


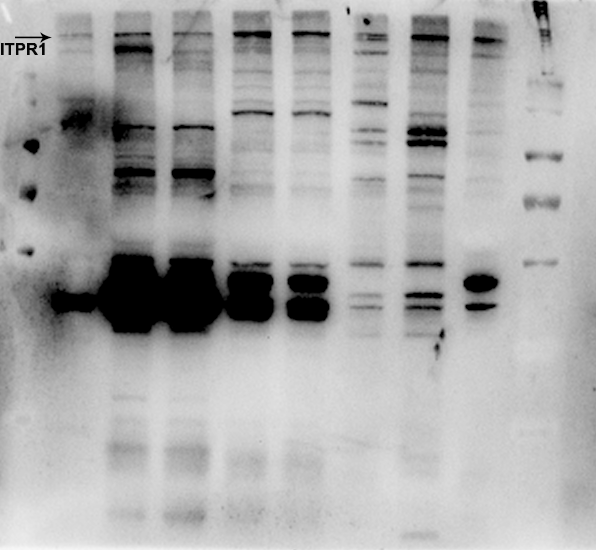

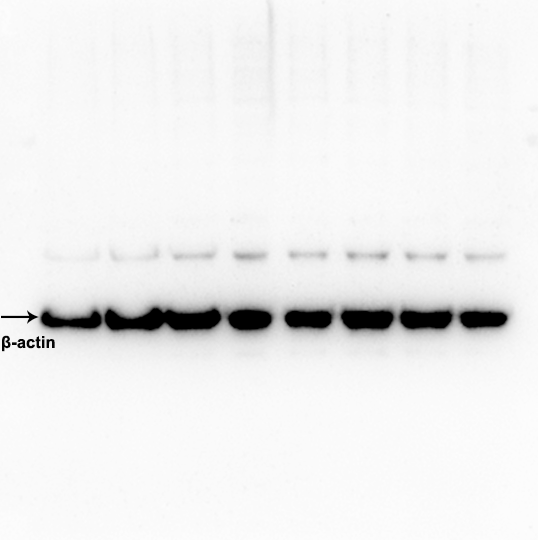

Supplement: Supplementary file 2 — Additional file 2. [file 12885_2022_9410_MOESM2_ESM.docx]
